# Aryl hydrocarbon receptor nuclear translocator limits the recruitment and function of regulatory neutrophils against colorectal cancer by regulating the gut microbiota

**DOI:** 10.1186/s13046-023-02627-y

**Published:** 2023-03-01

**Authors:** Yujing Bi, Qiuli Yang, Zhengchao Li, Yuexin Wang, Yufei Wang, Anna Jia, Zhiyuan Pan, Ruifu Yang, Guangwei Liu

**Affiliations:** 1 State Key Laboratory of Pathogen and Biosecurity, Beijing Institute of Microbiology and Epidemiology, 100071 Beijing, China; 2 Key Laboratory of Cell Proliferation and Regulation Biology, Ministry of Education, College of Life Sciences, Beijing Normal University, 100875 Beijing, China

**Keywords:** Tumor-associated neutrophils, ARNT, gut microbiota, Immune microenvironment, Colorectal cancer, microbiota

## Abstract

**Background:**

Although the role and mechanism of neutrophils in tumors have been widely studied, the precise effects of aryl hydrocarbon receptor nuclear translocator (ARNT) on neutrophils remain unclear. In this study, we investigated the roles of ARNT in the function of CD11b^+^Gr1^+^ neutrophils in colitis-associated colorectal cancer.

**Methods:**

Wild-type (WT), ARNT myeloid-specific deficient mice and a colitis-associated colorectal cancer mouse model were used in this study. The level and functions of CD11b^+^Gr1^+^ cells were evaluated by flow cytometry and confocal microscopy.

**Results:**

We found that ARNT deficiency drives neutrophils recruitment, neutrophil extracellular trap (NET) development, inflammatory cytokine secretion and suppressive activities when cells enter the periphery from bone marrow upon colorectal tumorigenesis. ARNT deficiency displays similar effects to aryl hydrocarbon receptor (AHR) deficiency in neutrophils. CXCR2 is required for NET development, cytokine production and recruitment of neutrophils but not the suppressive activities induced by *Arnt*^−/−^ in colorectal cancer. The gut microbiota is essential for functional alterations in *Arnt*^−/−^ neutrophils to promote colorectal cancer growth. The colorectal cancer effects of *Arnt*^−/−^ neutrophils were significantly restored by mouse cohousing or antibiotic treatment. Intragastric administration of the feces of *Arnt*^−/−^ mice phenocopied their colorectal cancer effects.

**Conclusion:**

Our results defined a new role for the transcription factor ARNT in regulating neutrophils recruitment and function and the gut microbiota with implications for the future combination of gut microbiota and immunotherapy approaches in colorectal cancer.

**Supplementary Information:**

The online version contains supplementary material available at 10.1186/s13046-023-02627-y.

## Background

Colorectal cancer is the third most common cancer and the second leading cause of cancer death worldwide [1]. Environmental, lifestyle and genetic factors are related to the occurrence of colorectal cancer [2, 3]. Recently, however, it was determined that imbalance of the gut microbiota and immunity was the cause of 90% of colorectal cancer [1, 4]. A large amount of epidemiological and experimental evidence has shown that chronic inflammation is crucial to the induction and progression of colorectal cancer [4, 5], but the underlying mechanism is still unclear. Chronic inflammation often leads to the continuous functional destruction of mucosal and gut microbiota protective barriers, which directly induce the mutation of intestinal epithelial cells and trigger the development and progression of colorectal cancer [6–8]. Therefore, in addition to chemotherapy or targeted drug therapy, immunotherapy and gut microbiota therapy are presently being considered for colorectal cancer patients [6]. However, there is still a lack of research on the regulatory mechanism and targeting strategy of immunity and the gut microbiota in the development of colorectal cancer, which greatly limits research on treatment strategies for colorectal cancer, especially colitis-associated colorectal cancer.

Neutrophils are the one of infiltrating immune cells in the tumor microenvironment that maintain tumor progression, especially in colitis-associated tumorigenesis [2, 9]. Previous studies have shown that CD11b^+^Gr1^+^ tumor-associated neutrophils are the main infiltrating immune cells of colitis-associated colorectal cancer [1, 9–11]. However, their precise immune regulation mechanism in colitis-associated tumorigenesis and their relationship with the gut microbiota have not been elucidated.

Aryl hydrocarbon receptor nuclear translocator (ARNT) is a transcription factor and belongs to the basic helix loop helix (bHLH)-per Arnt sim (PAS) family [12]. It is generally believed that ARNT plays a key role in two different fields: the aryl hydrocarbon receptor (AHR) and hypoxia inducible factor (HIF) pathways [13–15]. HIF1α and AHR have been shown to play important roles in tumorigenesis and proliferation, but the role of ARNT is still unclear. It is generally believed that its expression is constant, and it may not play a critical role [12]. However, recent studies suggest that ARNT may also play an important role, especially in tumor growth [13–15].

In this study, we found that ARNT deficiency in neutrophils, drives their recruitment, neutrophil extracellular trap (NET) formation, inflammatory cytokine secretion and suppressive activities in a gut microbiota-dependent manner in colitis-associated tumorigenesis.

## Materials and methods

### Mice

C57BL/6 *Arnt*^*fl/fl*^ and *Lyz-Cre* mice were obtained from the Jackson Laboratory and extensively backcrossed to the C57BL/6 background. Wild-type (WT) controls from ARNT knockout mice included Cre^+^ mice (*Lyz-Cre*) to account for the effects of *Cre*, as described previously [16–18]. Sex-matched littermates at 8 to 10 weeks of age were used in the experiments described in this study. All mice were bred and maintained under specific pathogen-free conditions. All experiments used cohoused littermates unless otherwise indicated so that consistency of common microbiota and genetic background/alterations would be ensured. All animal experimental protocols were approved by the Animal Ethics Committee of Beijing Institute of Microbiology and Epidemiology and Beijing Normal University (IACUC-DWZX-2017-003 and CLS-EAW-2017-002; Beijing, China). Animals were maintained in pathogen-free conditions.

### Tumor model and histological analysis

The colitis-associated colorectal cancer mouse model was established in our lab and other labs as described previously [19–21]. Mice of each genotype were given a single intraperitoneal administration of azoxymethane (AOM; Sigma–Aldrich, St. Louis, MO, USA; 10 mg/kg body weight). Five days later, these mice were randomly divided into two groups fed 2% dextran sulfate sodium salt (DSS; MP Biomedicals, Santa Ana, CA, USA) in drinking water for 3 cycles or plain water as a control. The experiment was terminated 80 days after the first injection of AOM. Mice were weighed and observed daily. Compared with baseline, the percentage of mouse weight loss was used to evaluate the severity of colitis, and the progress of the disease was observed in combination with daily observation of clinical symptoms such as rectal bleeding, diarrhea or prolapse. If there was a significant weight loss of more than 20% or obvious clinical symptoms, the experiment was terminated, and a humane end point was applied to the mice. As the AOM/DSS model is quite reliable in producing tumors, it is usually unnecessary to observe tumor growth with endoscopy, as described previously [21]. To establish subcutaneous tumors, 5 × 10^6^ MC38 colon cancer cells were injected into C57BL/6 mice, half male and half female, randomization group. These cells formed a tumor of 1–2 cm diameter within 2–3 weeks of injection and double blinding detecting of mouse tumor size, as described previously [22].

For neutrophils adoptive transfer experiments, CD11b^+^Gr1^+^ cells were isolated from femurs of AOM/DSS-treated WT mice by a Cell Isolation Kit (Miltenyi Biotec, Cologne, BG, Germany) after lysis of red blood cells (RBCs) according to the manufacturer’s instructions. The first flow through from the column contains unbound immune cells, which were used as controls (non-neutrophils). CD11b^+^Gr1^+^ cells were eluted twice, and the purity of CD11b^+^Gr1^+^ cells was more than 95% by flow cytometry. Isolated cells were subjected to neutrophil adoptive transfer experiments. A total of 5 × 10^6^ purified neutrophils was intravenously injected into one recipient mouse twice a week from the beginning of DSS treatment to the end of the experiments. To deplete CD11b^+^Gr1^+^ cells in vivo, 0.5 mg of depleting anti-Gr1 mAb (RB6-8C5; Biolegend, San Diego, CA, USA) was i.p. injected into the recipient mice on day − 1 before tumor induction, after which they were injected twice a week. To delete the mouse gut microbiota, broad-spectrum antibiotics were added to water, including 10 g/l ampicillin, 10 g/l neomycin sulfate, 10 g/l metronidazole, and 5 g/l vancomycin. The mixed antibiotic water was given by gavage in 0.5 ml once every two days.

At the end of the experiments, some colons from each group were used to count tumors and determine inflammation scores, and the rest were used to examine the profiles of immune cells. For histologic analysis, 4 μm thick sections from all groups were stained with hematoxylin and eosin (HE) to examine colonic inflammation and tumor morphology. Histological scoring of inflammation was determined as described previously [18, 19]. Briefly, inflammatory scores were graded by the amount of inflammation, the depth of inflammation, and the amount of crypt damage or regeneration. The unstained sections were subjected to immunostaining.

### Immunohistochemistry (IHC)

The colon or colorectal tumor tissues from mouse models were collected and fixed in 10% formalin overnight and embedded in paraffin. Formalin-fixed paraffin-embedded tissue was cut into 4 μm sections. The sections were stained using standard protocols for xylene and an alcohol gradient for deparaffinization. After antigen retrieval and unmarking procedures, the primary rat anti-mouse Gr1 antibody (1 ng/ml in 50 µl volume; RB6-8C5; Biolegend, San Diego, CA, USA) was incubated and stained. Sections were incubated with HRP goat anti-rat IgG antibody (2.0 µg/ml; Poly4054; Biolegend, San Diego, CA, USA) for 40 min and then developed with an Ultravision DAB Plus Substrate Detection System (TA-125-QHDX, Thermo Fischer Scientific, Waltham, MA, USA) for 2–5 min at room temperature, followed by hematoxylin staining, dehydration and coverslipping with Permount. Immunohistochemistry (IHC) slides were scanned with a Pannoramic Digital Slide Scanner (SDHISTECH, Budapest, Hungary), and images were cropped from virtual slides in Pannoramic Viewer.

### Neutrophil isolation and cell culture

At eighty days after the first AOM injection, the mouse colon was taken. Tumor tissue was carefully isolated, and the rest of the nontumor colon tissue was cut into pieces and washed with RPMI 1640. Then, the colon fragments were resuspended in a 2 ml solution of 1 mg/ml collagenase XI (Sigma–Aldrich, St. Louis, MO, USA) containing 20 U/ml DNase I (Sigma–Aldrich, St. Louis, MO, USA) and incubated at 37 °C for 30 min. PBS with 1% FBS and 5 mM ethylene diamine tetraacetic acid (EDTA) was used to neutralize digestion. Cells were washed twice (452 g, 5 min), resuspended in RPMI 1640, and filtered to remove clumps. Murine bilateral femurs and tibias were taken, and 10 ml syringes were used to flush the medullary cavity with RPMI 1640. After passing through the 200-mesh net, the RBCs were lysed and washed twice (452 g, 5 min), and the cells were resuspended in RPMI 1640. After peripheral blood was obtained from the retroorbital vein, the RBCs were lysed, washed twice and resuspended in RPMI 1640. The spleens were collected and ground. The RBCs were lysed and washed twice (452 g, 5 min), and the cells were resuspended in RPMI 1640. Mesenteric lymph nodes (MLNs) were taken, ground and washed twice (452 g, 5 min), and the cells were resuspended in RPMI 1640. Spleen and tumor tissues were dissected and digested with 0.7 mg/ml collagenase XI (Sigma–Aldrich, St. Louis, MO, USA) and 30 mg/ml type IV bovine pancreatic DNase (Sigma–Aldrich, St. Louis, MO, USA) for 45 min at 37 °C. Finally, single-cell suspensions were prepared from blood, spleen, BM, MLN, colon or tumor samples for further assay. CD11b^+^Gr1^+^ neutrophils were isolated from single-cell suspensions of the spleen, BMs, colon or tumor tissues by cell sorting on a FACSAria (BD Biosciences, Franklin Lake, NJ, USA), as previously described [10, 23].

BM-derived CD11b^+^Gr1^+^ cells were generated as described previously [24–26]. In brief, BM cells were cultured in complete DMEM supplemented with 2 mM l-glutamine, 10 mM HEPES, 20 mM 2-ME, 150 U/ml streptomycin, 200 U/ml penicillin, and 10% FBS and stimulated with combinations of recombinant murine GM-CSF (10 ng/ml, Peprotech, Rocky Hill, NJ, USA). The cultures were maintained at 37 °C in a 5% CO_2_-humidified atmosphere for 4 days. Normal human peripheral blood neutrophils (915,410, Beijing Nuowei Biology, Beijing, China) were treated by GNF351 (50–500 nM, MCE) and stimulated for 12 h with LPS for further analysis.

### Immunosuppressive activities assay

CD11b^+^Gr1^+^ cells were sorted from the spleen, BM, colon or tumor tissues by flow cytometry. The suppressive activities of neutrophils were assessed by determining their abilities to inhibit T-cell proliferation as described previously [16, 27]. C57BL/6 CD4^+^ T cells (1 × 10^5^ cells/well) were cocultured with 15 µg/ml mitomycin C (Sigma–Aldrich, St. Louis, MO, USA)-pretreated BALB/c splenocytes (1 × 10^5^ cells/well), and neutrophils (1 × 10^5^ cells/well) were sorted at different ratios in a flat-bottom 96-well plate at 37 °C in 5% CO_2_. Cell proliferation was determined 72 h after incubation with [^3^ H] thymidine for the last 16 h of culture.

### Flow cytometry

Single-cell suspensions were prepared from the blood, spleen, BMs, colon or tumor tissues. For flow cytometric analysis of cell surface markers, cells were stained with mAbs in PBS containing 2% (w/v) BSA and 0.1% NaN_3_ for 30 min at 4 °C, as described previously [28]. The following mAbs were obtained from eBioscience (San Diego, CA, USA): FITC rat anti-mouse CD11b (M1/70), PE rat anti-mouse CD11b (M1/70), APC rat anti-mouse CD11b (M1/70), PE rat anti-mouse Gr1 (RB6-8C5), APC rat anti-mouse Gr1 (RB6-8C5), FITC rat anti-mouse Gr1 (RB6-8C5), FITC rat IgG 2b (eB149/10H5), PE rat IgG 2b (eB149/10H5), and APC rat IgG 2b (eB149/10H5). The following mAbs were obtained from Biolegend (San Diego, California, USA): PE/Cyanine7 rat anti-mouse CD45 (30-F11). The following antibodies were obtained from R&D system (Minnesota, USA): PE rat anti-mouse CXCR2 (242,216), APC rat anti-mouse CXCR2 (242,216), PE rat anti-mouse CD115 (460,615), PE mouse anti-human CXCR2 (48,311), PE rat IgG2a (54,447), APC rat IgG2a (54,447) and PE mouse IgG2a (20,102). The following antibodies and reagents were obtained from Abcam (Cambridge, UK): APC rat anti-mouse Ly6G (RB6-8C5), FITC rat anti-mouse Ly6C (HK1.4), FITC rat IgG2c (RTK4174), and 7-AAD staining solution.

FACS-based intracellular staining of cytokines was performed as previously described [28]. Cells were stimulated with LPS (100 ng/ml, Sigma–Aldrich, St. Louis, MO, USA) and GolgiPlug (BD Pharmingen, Lake Franklin, NJ, USA) for 5 h. BD Cytofix/Cytoperm and BD Perm/Wash buffer sets were used according to the manufacturer’s instructions (BD Pharmingen, Lake Franklin, NJ, USA). APC rat anti-mouse IL-10 (JES5-16E3) and its APC rat IgG2b isotype control (RTK4530); APC rat anti-mouse tumor necrosis factor α (TNFα; MP6-XT22) and its APC rat IgG1 isotype control (RTK2071) were obtained from Biolegend (San Diego, CA, USA). The same amount of isotype control staining used as the negative control.

Flow cytometry data were acquired on an ACEA NovoCyte (ACEA Biosciences, Inc., San Diego, CA, USA), and data were analyzed with NovoExpress or Flow Jo (TreeStar, San Carlos, CA, USA). The viability dye was used to exclude dead cells and isotype matched immunoglobulins staining was performed to set up the regions and quadrants of the negative control, and then the percentages of antibody staining of various targets were determined.

### Quantitative RT–PCR

RNA was extracted with a RNeasy kit (QIAGEN, Dusseldorf, Germany), and cDNA was synthesized using SuperScript III reverse transcriptase (Invitrogen, Carlsbad, CA, USA). An ABI 7900 real-time PCR system was used for quantitative PCR, with primer and probe sets (Supplementary Table 1) obtained from Applied Biosystems (Carlsbad, CA, USA). The results were analyzed using SDS 2.1 software (Applied Biosystems, Foster City, CA, USA). The expression of each target gene is presented as the fold change relative to that of control samples, as described previously [22, 28].

### AHR knockdown with RNA interference

A gene-knockdown lentiviral construct was generated by subcloning gene-specific short hairpin RNA (shRNA) sequences into lentiviral shRNA expression plasmids (pLL3.7). The AHR shRNA sequence was 5′-AAG UCG GUC UCU AUG CCG CTT-3′, and the control shRNA sequence was 5′-GCG CGC UUU GUA GGA UUC GTT-3′. Lentiviruses were harvested from the culture supernatant of 293T cells transfected with shRNA vector. BM cells were cultured in complete DMEM supplemented in the presence of recombinant murine GM-CSF for 4 days to induce CD11b^+^Gr1^+^ neutrophils. Neutrophils were infected with recombinant lentivirus, and GFP-expressing cells were isolated using fluorescence sorting 48 h later. AHR expression was confirmed using qPCR. The sorted CD11b^+^Gr1^+^ cells with control or shRNA vectors were used for further assays.

### Retroviral transduction of ARNT

Retroviral transduction ARNT were cloned into the MSCV retroviral vector (Clontech Laboratories, Mountain View, CA), as previously described [25]. Phoenic-Eco packaging cells (ABP-RVC-10,001; Allele Biotechnology, San Diego, CA) were transfected with Lipofectamine (Invitrogen), and recombinant retrovirus was collected 48 h after transfection. After 2 days of differentiation, BM cells were cultured in complete DMEM supplemented in the presence of recombinant murine GM-CSF for 4 days to induce CD11b^+^Gr1^+^ neutrophils. Neutrophils were transduced with retroviral supernatant by spin inoculation and GFP-expressing cells were isolated by flow cytometry and performed the further analysis.

### Western blot

BM cells were cultured in complete DMEM in the presence of recombinant murine GM-CSF for 4 days to induce CD11b^+^Gr1^+^ neutrophils. The sorted CD11b^+^Gr1^+^ cells were washed twice with cold PBS and lysed in RIPA buffer (50 mM Tris-HCL, pH 7.4, 1% NP-40, 0.25% Na-deoxycholate, 150 mM NaCl, 1 mM EDAT, pH 7.4) for 10 min on a rocker at 4 °C. The protein concentration was determined via bicinchoninic acid assay (BCA; Beyotime, Shanghai, China). The protein samples were separated by 10% SDS–PAGE and then transferred onto 0.22 μm polyvinylidene fluoride membranes (Merck Millipore, Bedford, MA, USA). The membranes were blocked with 5% nonfat dried milk for 1 h at room temperature and incubated with rabbit anti-mouse primary antibodies (1:200) overnight on a shaker at 4 °C. Subsequently, HRP-coupled secondary goat anti rabbit antibody (1:10000; Beyotime, Shanghai, China) was added for 1 h at room temperature. After sufficient washing, protein samples were detected with an eECL Western Blot Kit (Cat: CW0049M, CWBIO, Taizhou, Jiangsu, China) using AllDoc-x software with a Tanon 5200 Imager (Tanon, Shanghai, China). The following primary Abs were used: anti-ARNT (D28F3) was obtained from Cell Signaling Technology (Danvers, MA, USA), anti-β-actin (AC-15) was obtained from Sigma–Aldrich (St. Louis, MO, USA), anti-AHR (A3) and anti-CYP1A1 (B4) were obtained from Santa Cruz Biotechnology (Santa Rosa, CA, USA), anti-cyclooxygenase (COX) 2 was obtained from Abcam (Cambridge, UK), and anti-CYP1B1 (K008135P) and anti-AHRR (K007675P) were purchased from Beijing Solarbio Science & Technology Company (Beijing, China).

### ELISA

Colorectal cancer induction for 80 days after the injection of AOM and the addition of DSS to the drinking water as described in the tumor model section. The concentrations of serum CXCL1 and CXCL2 at day 80 were quantified by sandwich ELISA. Before execution, fresh mouse feces were collected and partly resuspended with PBS. Fecal supernatant was used to stimulate RAW264.7 cells for 3 h, and the concentrations of TNFα and IL-1β in the culture supernatant were detected by sandwich ELISA. Mouse CXCL1 (MKC00B) and CXCL2 (MM200) ELISA kits were obtained from R&D Systems (Minneapolis, MN, USA), and mouse IL-1β (#abs520001), mouse TNFα (#abs552812), human TNFα (#abs510006) and human IL-10 (#abs510005) ELISA kits were obtained from Absin Biotechnology Co., Ltd (Shanghai, China), as described previously [22].

### Fecal collection and 16 S rRNA gene sequencing

Colorectal cancer induction for 80 days after the injection of AOM and the addition of DSS to the drinking water. At day 80, fresh stool pellets were obtained and immediately frozen at -80 °C. Fecal DNA was extracted from the feces using the QIAamp DNA Stool Mini Kit (Qiagen, Hilden, Germany) according to the manufacturer’s instructions.

The V4 region of the 16 S rRNA gene was selected as the sequencing region to compare the diversity and structure of the bacterial species in each of the samples. The primers for the V4 region were 515 F (GTGCCAGCMGCCGCGGTAA) and 806R (GGACTACHVGGGTWTCTAAT). Sequencing was performed by Illumina MiSeq at QualityHealth Bioinformatics Technology Co., Ltd. (Beijing, China).

### Sequence analysis

Paired-end reads 250 bp in length in each direction were generated, and the overlapping reads were stitched together. The raw paired-end reads were assembled using Pandaseq. A self-written script was run to discard low-quality reads with an average quality score < 20, containing > 3 nitrogenous bases, and with lengths of < 220 or > 500 nucleotides. The high-quality sequences were then treated to filter singletons and remove chimeras.

The 16 S rDNA sequence data were analyzed using UPARSE software (UPARSE v7.0.1001, http://drive5.com/uparse/), which divided the sequences into OTUs using a similarity threshold of 97%. A similarity score of < 97% was considered indicative of a different species, and a score of < 93–95% was considered indicative of a different genus. The SSUrRNA database was used to annotate the species, and MUSCLE (Version 3.8.31, http://www.drive5.com/muscle/) was used to BLAST the sequences. Finally, all data were normalized for further analysis.

Diversity within samples (α-diversity) and between samples (β-diversity) was estimated on the basis of the OTUs. α-Diversity refers to the diversity of a particular region or ecosystem and is an expression of the species diversity in a single sample. β-Diversity indices were used to estimate the distance between samples based on the evolutionary relationship of the sample sequence and its abundance. β-Diversity indices are expressed in terms of the differences between the sample groups by means of principal coordinate analysis (PCoA). Metastat and LDA effect size (LefSe) analyses were used to detect significant differences among groups in the microbial community biomarkers.

### RTCA for fecal metabolite analysis

Fresh stool pellets were collected from colorectal cancer model mice at the day 80, and fecal supernatant was obtained after feces were resuspended in PBS. All the fecal supernatant was filtered. Real-time cellular analysis (RTCA) was used to assess the effect of fecal metabolites on intestinal epithelial cells. Briefly, HT-29 cells (6 × 10^4^/well) were plated on E-plates, then put the E-plates into XCELLigene (ACEA Bioscience, USA), which was set the automatic detection interval every 5 min. Five hours later, fecal filtrate (100 µl/well) was added to E-plates. The E-plates were detected for 20 h. The cell response curves and IC50 values of different time periods can be obtained by continuous real-time dynamic detection.

### NET formation assays

Neutrophils were sorted from the single-cell suspensions of the BM or tumor tissue. A total of 2 × 10^5^ cells was plated in 200 µl in a 96-well flat bottom plate and incubated for the indicated time, and 5% CO_2_ and Sytox Green (25 nM, Thermo Fisher, Waltham, MA, USA) were added and incubated for 5 min. Cells were fixed with 16% paraformaldehyde (PFA) and kept at 4 °C until confocal microscopy was performed to quantify NET formation. Z-Stacks (10–30 μm 40x magnification) were taken using an LSM800 equipped with a 488 diode and a Plan-Apochromat 1,3 N/An Oil DIC III objective. For NET area quantification, FIJI software and the particle analysis plugin were used. Only structures depicting NET morphology and positive for Sytox green were selected for area quantification, and intact granulocyte nuclei were excluded from the analysis. Triplicate wells of each condition were included.

### Statistical analysis

All data are presented as the mean ± SD. Student’s unpaired *t* test was used to compare two sets of parametric data. When comparing three or more datasets, one-way analysis of variance with Dunnett’s post-hoc test was applied for parametric data, and a Kruskal–Wallis test was applied for nonparametric data. A comparison of survival curves was performed using the log-rank (Mantel–Cox) test. A *P* value of less than 0.05 was considered statistically significant.

## Results

### ARNT expressions in neutrophils

**Fig. 1:**
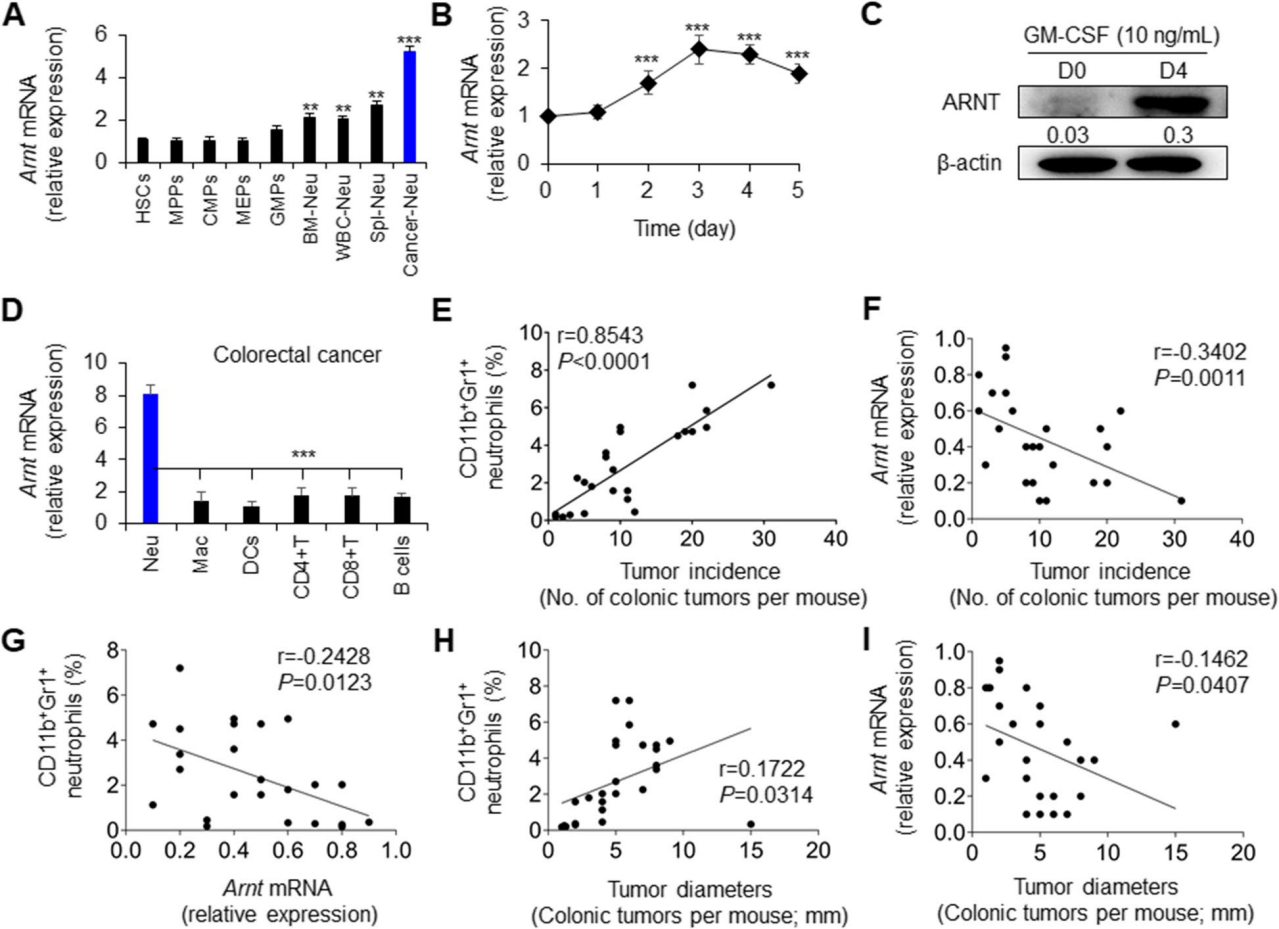
Colorectal tumorigenesis is related to neutrophil level and ARNT expression. **A** Quantitative PCR analysis of *Arnt* mRNA expression during neutrophil development from HSCs. The graphs summarize data from two independent experiments, each with six mice. **B-C** Quantitative PCR analysis (**B**) and western blot (**C**) of ARNT expression in neutrophils induced by GM-CSF for 4 days from BMs. The graphs summarize data from two independent experiments, each with six mice (**B**). Representative of two independent experiments (**C**). **D-I** Colorectal cancer induction for 80 days after the injection of azoxymethane (AOM) and dextran sulfate sodium salt (DSS). (**D**) Quantitative PCR analysis of *Arnt* mRNA expression in different subpopulations of tumor-infiltrating immune cells, including neutrophils (Neu; CD11b^+^Gr1^+^ cells), macrophages (Mac; CD11b^+^F4/80^+^ cells), DCs (CD11c^+^ cells), CD4^+^T cells (CD4^+^TCR^+^ cells), CD8^+^T cells (CD8^+^TCR^+^ cells) and B cells (CD19^+^ cells) isolated from tumors. Representative of three independent experiments with four mice. The correlations between the occurrence of tumors, the level of neutrophils and the expression of *Arnt* (**E**-**G**) and the correlations between the level of neutrophils, the expression of *Arnt* and the growth of tumors were analyzed (**H-I**), as indicated in the figure. The graph summarizes data from four independent experiments, each with five to six mice **(E-I**). ***P < 0.001, compared with the indicated groups

To evaluate the effects of ARNT on the development and function of neutrophils, we determined the expressions of ARNT-mRNA through myeloid development in a highly purified population from BM to peripheral blood or spleen after excluding dead cells by viability staining (Fig. S1A). The data showed that there were no significant alterations in the expression of ARNT-mRNA in hematopoietic stem cells (HSCs), multipotent progenitors (MPPs), common myeloid progenitors (CMPs) and granulocyte-monocyte progenitors (GMPs) before the development and differentiation of mature neutrophils, but ARNT-mRNA expression in mature neutrophils in BMs released into the blood and spleen increased significantly (Fig. 1A). Furthermore, in the BM neutrophil culture system in vitro, ARNT-mRNA expression was progressively enhanced in CD11b^+^Gr1^+^ neutrophils (Fig. 1B-C). These results suggest that ARNT may be involved in the development and functional regulation of neutrophils.

Importantly, in colorectal cancer mice, the expression of ARNT was highest in tumor-infiltrating neutrophils among various immune-infiltrating cells (Fig. 1D). Moreover, the expression of ARNT-mRNA in tumor-infiltrating neutrophils was higher than in mature neutrophils under physiological conditions (Fig. 1A). Further analysis showed that the level of neutrophils was positively correlated with the number of colonic tumors and their size, but the expression of ARNT was negatively correlated with the number of colonic tumors and their size, and the level of tumor-infiltrating neutrophils was negatively correlated with the expression of ARNT (Fig. 1E-I). It is further suggested that the expression of ANRT in neutrophils might be related to the level of tumor-infiltrating neutrophils, tumorigenesis and growth.

### ***Arnt***^−/−^promotes the migration and function of neutrophils

**Fig. 2:**
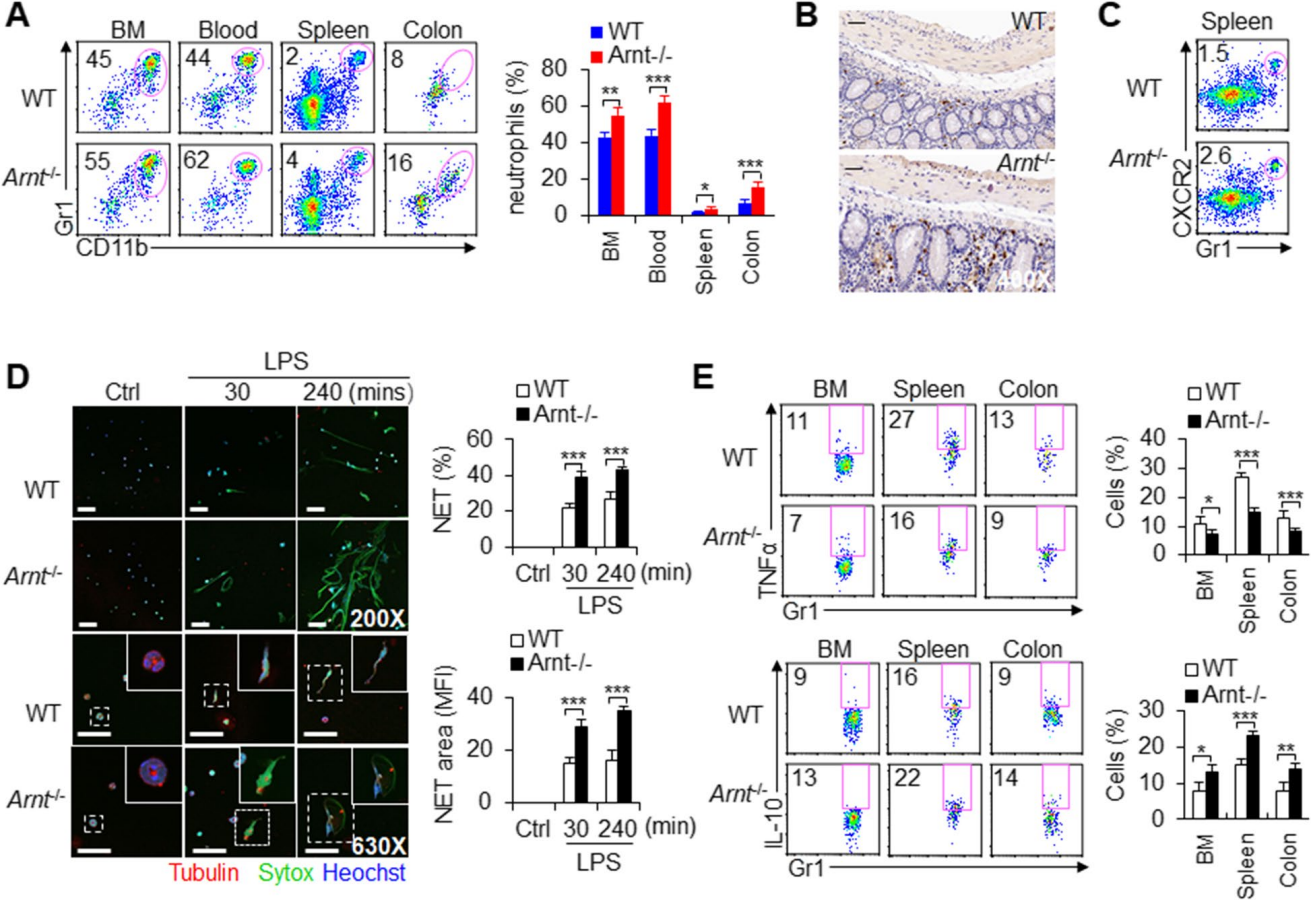
***Arnt***^−/−^enhanced the migration and functions of neutrophils. **A** *Arnt*^−/−^ enhanced the percentage of CD11b^+^Gr1^+^ neutrophils in BMs, blood, spleen and colon. Dot-plots present the representative data from flow cytometry analysis is shown (left), and the data are summarized (right). The graph summarizes data from two independent experiments with six mice per group. **B** Immunohistochemistry analysis of colon after staining with anti-Gr1 antibody. Scales bars, 50 μm. Original magnification, 400X. Representative of three independent experiments. **C** CXCR2 expression in neutrophils and dot-plots present the representative data from flow cytometry analysis is shown. Representative of three independent experiments. **D** Neutrophils from BMs were isolated and stimulated with LPS in vitro as indicated to detect neutrophil extracellular traps (NETs). Typical NET images are displayed (left), and the percentage and area of NETs are quantified (right). Scales bars, 20 μm (upper) and 50 μm (lower). Original magnification, 200X (upper) and 630X (lower). The graph summarizes data from three independent experiments with six mice per group. **E** The intracellular staining of TNFα and IL-10 in neutrophils was detected by flow cytometry. Dot-plots present the representative data from flow cytometry analysis is shown (left), and statistical results are shown (right). The graph summarizes data from three independent experiments with six mice per group. *, *P* < 0.05; **, *P* < 0.01 and ***P < 0.001, compared with the indicated groups

To test this hypothesis, we crossed *Arnt*^flox/flox^ mice with *LysM*-*Cre* mice and deleted ARNT in myeloid cells, hereafter named *Arnt*^−/−^ mice. Although there was no significant change in colon appearance or microscopic morphology (Fig. S1B-C), *Arnt*^−/−^ mice retained more CD11b^+^Gr1^+^ neutrophils in BM, blood, spleen and colon tissue than WT mice (Fig. 2A-B). These results suggest that *Arnt*^−/−^ probably promotes the migration of neutrophils. CXCR2 is an important chemotactic receptor of myeloid cells that often mediates the migration and recruitment of neutrophils [5, 23]. The percentages and mean fluorescence intensity (MFI) of CXCR2 also increased significantly in *Arnt*^−/−^ neutrophils (Figs. 2C and S1D). Therefore, these data collectively suggest that *Arnt*^−/−^ probably promotes neutrophil migration.

We further evaluated the function of neutrophils, including NET formation and cytokine secretion in neutrophils. BM neutrophils were stimulated with LPS for 30 and 240 min to observe the formation of NETs. *Arnt*^−/−^ promoted the formation of NETs (Fig. 2D). *Arnt*^−/−^ significantly inhibited the production of the proinflammatory factor TNFα and promoted the production of the anti-inflammatory cytokine IL-10 (Fig. 2E). These data suggest that ARNT deficiency might promote the immunosuppressive activity of neutrophils.

To exclude the effect of cell compensatory regulation in vivo, we isolated BM cells and stimulated them with GM-CSF for 4 days to induce CD11b^+^Gr1^+^ cells in vitro. The results showed that *Arnt*^-/-^ significantly promoted the number of neutrophils, NET formation, IL-10 secretion and CXCR2 expression (Fig. S2A-D). Additionally, the production of CXCL1 and CXCL2, but not CCL3 or CCL5, chemokines that mediate neutrophil migration, increased significantly in the *Arnt*^-/-^ groups compared with the WT compartment (Fig. S2E). These results are similar to the AHR deficiency in neutrophils. AHR knockdown in neutrophils promoted NET formation, IL-10 production and CXCR2 expression (Fig. S2F-I). However, overexpression of ANRT in neutrophils reduced the NET formation, induced more proinflammatory factor TNFα and fewer anti-inflammatory factor IL-10 production and lower suppressive activities (Fig. S3A-D). Importantly, ARNT deficiency did not affect the expression of AHR signaling molecules, including AHR, CYP1A1, CYP1B1 and COX2, in neutrophils (Fig. S4). These data collectively showed that ARNT deficiency has similar effects to AHR deficiency in neutrophils which is based most likely on a disrupted canonical AHR signaling pathway by the lack of its dimerization partner ARNT.

### ANRT deficiency potentiated the recruitment and function of neutrophils during colorectal cancer growth

**Fig. 3:**
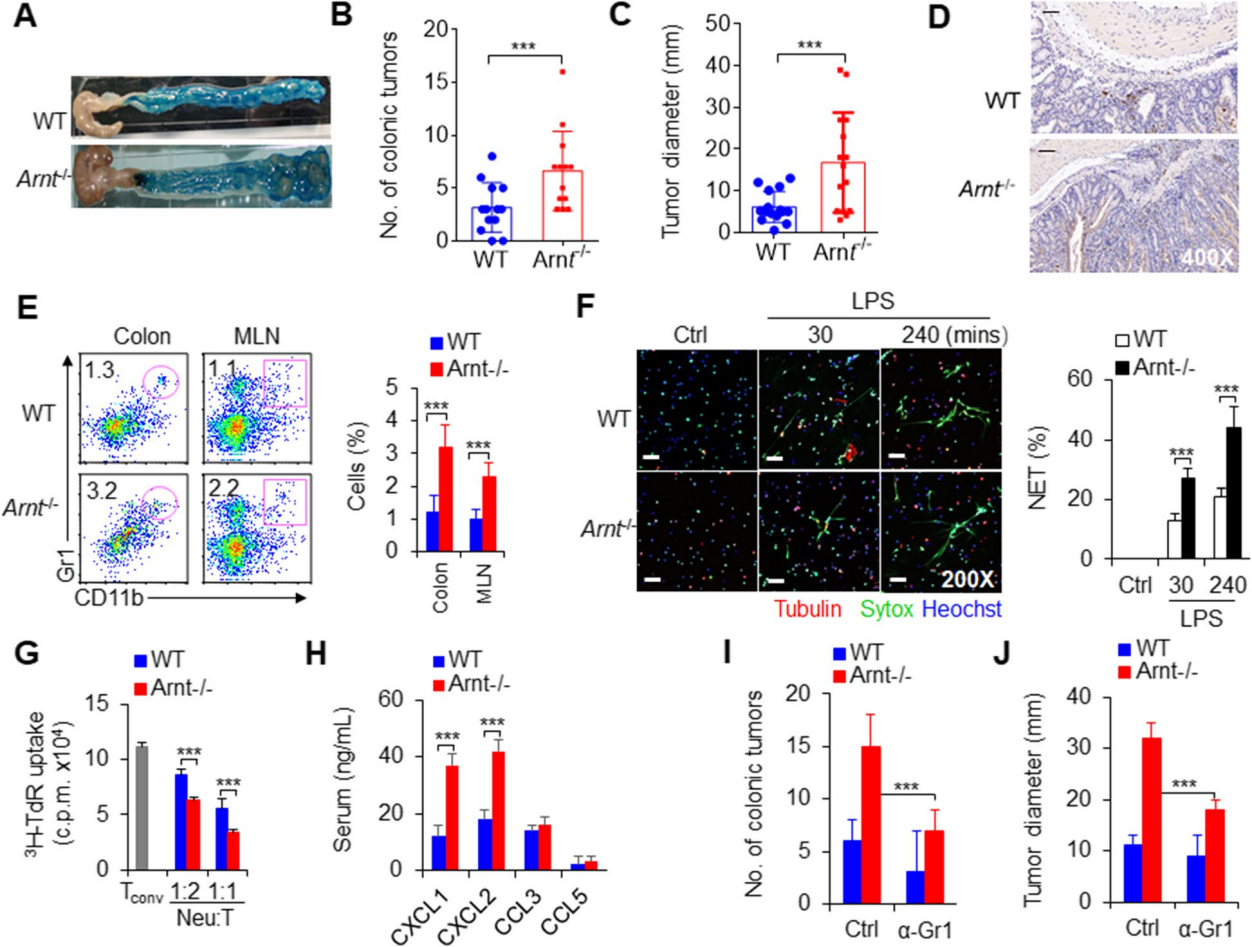
The***Arnt***^−/−^tumor microenvironment enhances neutrophil recruitment and functions in promoting colorectal cancer growth. Colorectal cancer induction for 80 days after the injection of azoxymethane (AOM) and the addition of dextran sulfate sodium salt (DSS) to the drinking water. **A** A typical photo of colorectal cancer. Representative of three independent experiments. **B** Summary of the number (No.) of colonic tumors. The graph summarizes data from three independent experiments with thirteen mice per group. **C** Summary of tumor diameter. The graph summarizes data from three independent experiments with thirteen mice per group. **D** Immunohistochemistry analysis of colorectal cancer after staining with anti-Gr1 antibody. Scales bars, 50 μm. Original magnification, 400X. Representative of three independent experiments. **E** Percentages of neutrophils in the colon and MLN. Dot-plots present the representative data from flow cytometry analysis is shown on the left, and the data are summarized on the right. The graph summarizes data from three independent experiments with six mice per group. **F** Tumor-infiltrating neutrophils were isolated and stimulated with LPS in vitro as indicated to detect NETs. Typical NET images are displayed (left), and the percentage of NETs-forming cells is quantified (right). Scales bars, 20 μm. Original magnification, 200X. The graph summarizes data from three independent experiments with six mice per group. **G** Suppressive assay of tumor-infiltrating neutrophils from tumor-bearing WT and *Arnt*^−/−^ mice. Data from coculture with allogeneic CD4^+^ T cells with different ratio between neutrophils and T cells are shown. Representative of three independent experiments. **H** Indicated chemokine production in serum from tumor-bearing WT and *Arnt*^−/−^ mice. Representative of three independent experiments. **I-J** Effects of neutrophils injected with anti-Gr1 mAb on colorectal cancer incidence (**I**) and diameter (**J**). The graph summarizes data from three independent experiments with twelve mice per group. ***P < 0.001, compared with the indicated groups

To investigate the biological significance of ARNT deletion in CD11b^+^Gr1^+^ neutrophils, we used the combination of AOM and DSS to induce spontaneous colorectal cancer development. ARNT deletion significantly promoted the occurrence and number of colorectal tumors, although it did not significantly change the weight of mice (Figs. 3A-C and S5A). However, immunohistochemical staining of colon tissue showed that neutrophil infiltration increased significantly (Fig. 3D). Consistently, flow cytometry analysis showed that the amount of tumor CD11b^+^Gr1^+^ neutrophil infiltration increased significantly, mostly CD11b^+^Gr1^+^Ly6G^hi^CD115^−^ cells but not CD11b^+^Gr1^+^Ly6C^hi^CD115^+^ cells (Figs. 3E and S5B). ARNT-deficient neutrophils exhibited significantly more NET formation (Figs. 3F and S5C), lower proinflammatory factor secretion (Fig. S6A-B), and upregulated immunosuppressive activity (Fig. 3G). Consistently, the production and expression of the chemokines CXCL1 and CXCL2, but not CCL3 and CCL5, were significantly upregulated in serum and tumor-infiltrating neutrophils from *Arnt*^−/−^ mice (Fig. 3H and Fig. S6C). Additionally, the MFI of CXCR2 in neutrophils increased significantly in *Arnt*^−/−^ mice compared with WT mice (Fig. S6D). However, there were no significant changes in the expression levels of AHR signaling pathway-related molecules, including AhR, AhRR, Cyp1a1, Cyp1a2, Cyp1b1 and Cox2, in tumor-infiltrating neutrophils from *Arnt*^−/−^ mice (Fig. S6E). Next, we studied the effects of ANRT deficient neutrophils in MC38 mouse colon cancer. We observed changes in tumor growth in *Arnt*^−/−^ and WT mice. The rate of tumor growth was significantly faster and greater in *Arnt*^−/−^ than in WT mice (Fig. S7A-B). *Arnt*^−/−^ mice had more CD11b^+^Gr1^+^ neutrophil infiltration, fewer proinflammatory factor TNFα production, more anti-inflammatory factor IL-10 production and CXCR2 expression in neutrophils in tumor and draining lymph node (dLN) compared with WT control (Fig. S7C-F). These findings collectively indicate that tumor-infiltrating *Arnt*^−/−^ neutrophils, probably contribute to colorectal cancer growth.

To ascertain the role of neutrophils in tumor growth, in the process of colorectal cancer induction, we used an anti-Gr1 antibody injection to eliminate neutrophils every week to observe the effect of colorectal cancer growth. Surprisingly, the anti-Gr1 antibody injection significantly removed the tumor-infiltrating neutrophils, decreased the number of colorectal tumors and delayed the growth of colorectal cancer (Fig. 3I-J). Furthermore, we adoptively transferred WT and *Arnt*^−/−^ neutrophils into recipient WT mice twice a week and then induced colorectal cancer with AOM and DSS in recipient WT mice. The incidence and diameter of colorectal cancer in recipient WT mice treated with *Arnt*^−/−^ neutrophils were significantly increased compared with those in the WT control compartment (Fig. S8A-C). Collectively, these results suggest that neutrophils play a crucial role in the development and growth of colorectal cancer through ARNT signaling.

### Blocking CXCR2 limits neutrophil recruitment and NET formation in ***Arnt***^−/−^mice

Recently, CXCR2, a chemokine receptor, has been shown to play a key role in regulating NET formation of neutrophils in addition to accumulating neutrophils [9, 24]. Although the absence of ARNT significantly promotes the expression of CXCR2 in neutrophils of BMs and spleen under physiological conditions and pathological tumor-infiltrating neutrophils, can CXCR2 mediate the regulation of ARNT deficiency on neutrophil function? To determine this, we further evaluated the regulatory role of CXCR2 in ANRT-deficient neutrophils.

**Fig. 4:**
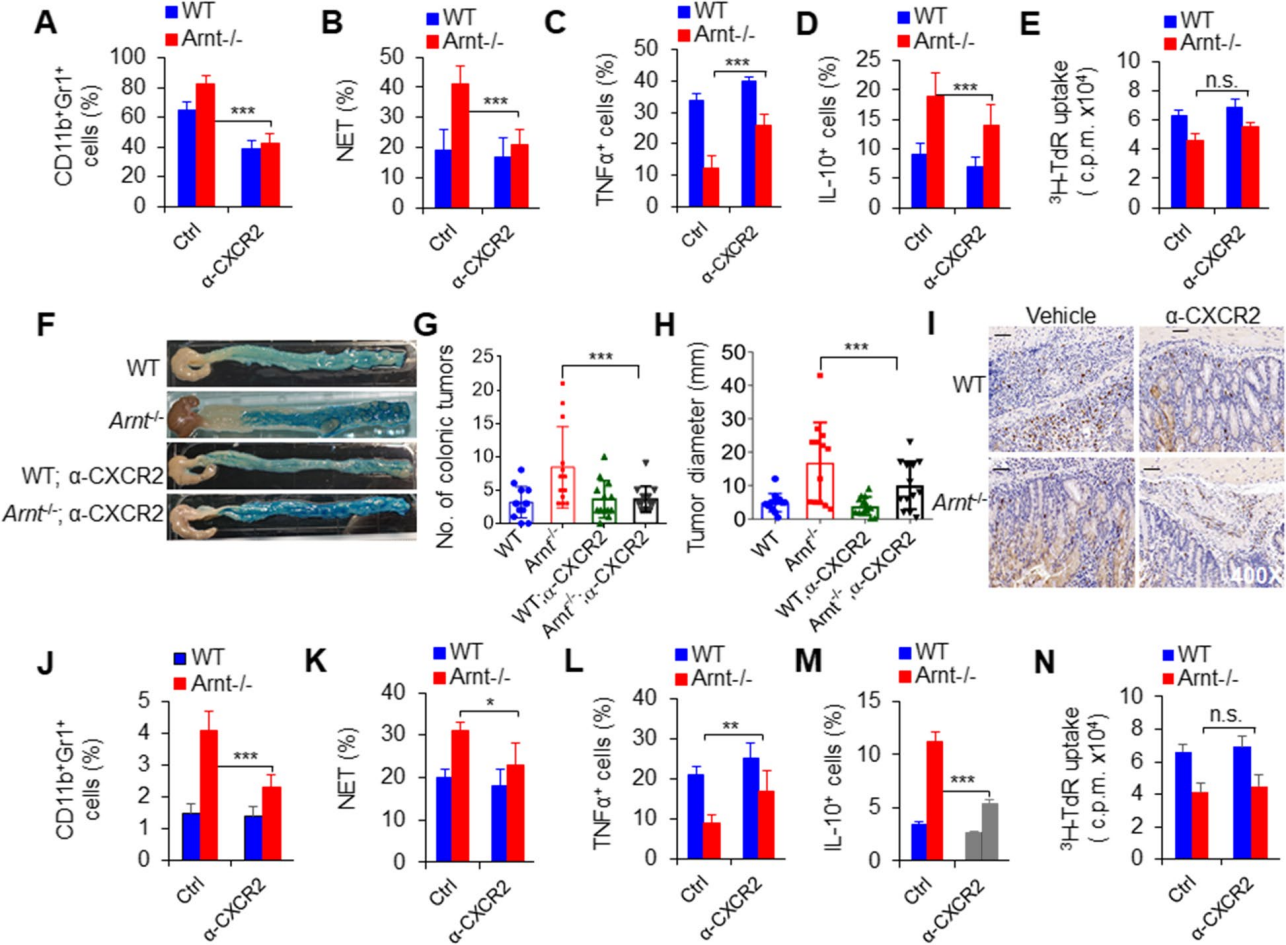
Blocking CXCR2 significantly restored ***Arnt***^−/−^-induced neutrophil recruitment and NET formation in colorectal cancer. **A-E** Neutrophils induced by GM-CSF from BMs in the presence of anti-CXCR2 for 4 days in vitro. Percentage of CD11b^+^Gr1^+^ neutrophils (**A**), percentage of NETs in neutrophils (**B**), intracellular staining of TNFα (**C**) and IL-10 (**D**) in neutrophils, suppressive activity assay of neutrophils (**E**). Representative of three independent experiments. **F-N** Colorectal cancer induction for 80 days after the injection of azoxymethane (AOM) and the addition of dextran sulfate sodium salt (DSS) to the drinking water and weekly anti-CXCR2 injection. **F** Typical photos of colorectal cancer. Representative of three independent experiments. **G** Summary of the number of colonic tumors. The graph summarizes data from three independent experiments with thirteen mice per group. **H** Summary of tumor diameter. The graph summarizes data from three independent experiments with thirteen mice per group. **I** Immunohistochemistry analysis of colorectal cancer after staining with anti-Gr1 antibody. Scales bars, 50 μm. Original magnification, 400X. Representative of two independent experiments. **J** Percent of neutrophils in the colon. **K** Tumor-infiltrating neutrophils were isolated and stimulated with LPS in vitro as indicated to detect NETs. The percentage of NETs-forming cells was quantified. **L-M** Intracellular staining of TNFα (**L**) and IL-10 (**M**) in neutrophils from the colon. **N** Suppression assay of tumor-infiltrating neutrophils. The graph summarizes data from three independent experiments with at least four mice per group (**J**-**N**). *, *P* < 0.05; **, *P* < 0.01 and ***P < 0.001, compared with the indicated groups

To directly test the regulatory effects of ARNT on neutrophils under physiological conditions, we used an in vitro BM neutrophil culture system. The results showed that *Arnt*^−/−^ significantly promoted the percentage of CD11b^+^Gr1^+^ cells, NET formation, and immunosuppressive activities of neutrophils and altered the cytokine production of neutrophils. However, blocking CXCR2 significantly recovered the percentage of CD11b^+^Gr1^+^ cells, NET formation and cytokine secretion but not the immunosuppressive activities of neutrophils (Fig. 4A-E). These data suggest that CXCR2 is necessary for *Arnt*^−/−^ to direct the expansion, NET formation and cytokine production of neutrophils but not their immunosuppressive activities.

Furthermore, in the tumor microenvironment, the role of CXCR2 in ARNT-deficient colorectal cancer mice was evaluated. In the combined treatment of AOM and DSS for the induction of colorectal cancer, a CXCR2-blocking antibody was injected every other week to block the expression of CXCR2, and the growth of mouse colorectal cancer was observed. *Arnt*^−/−^ mice had significantly more and larger colorectal tumors than WT mice, but blocking CXCR2 with an anti-CXCR2 mAb significantly decreased the incidence of colorectal cancer and delayed the growth of colorectal cancer in mice (Fig. 4F-H). Consistently, *Arnt*^−/−^ mice demonstrated significantly enhanced infiltrating neutrophil amounts, NET formation and cytokine production compared with WT controls, but blocking CXCR2 significantly recovered these alterations induced in *Arnt*^−/−^ mice (Fig. 4I-M). Of note, although *Arnt*^−/−^ enhanced the suppressive activities of neutrophils, blocking CXCR2 did not alter the effects (Fig. 4N). Therefore, CXCR2 signaling is required for ARNT-directed neutrophil recruitment and function while contributing to colorectal cancer development and growth.

### Neutrophils ***Arnt***^−/−^ alters the gut microbiota in colorectal cancer

**Fig. 5:**
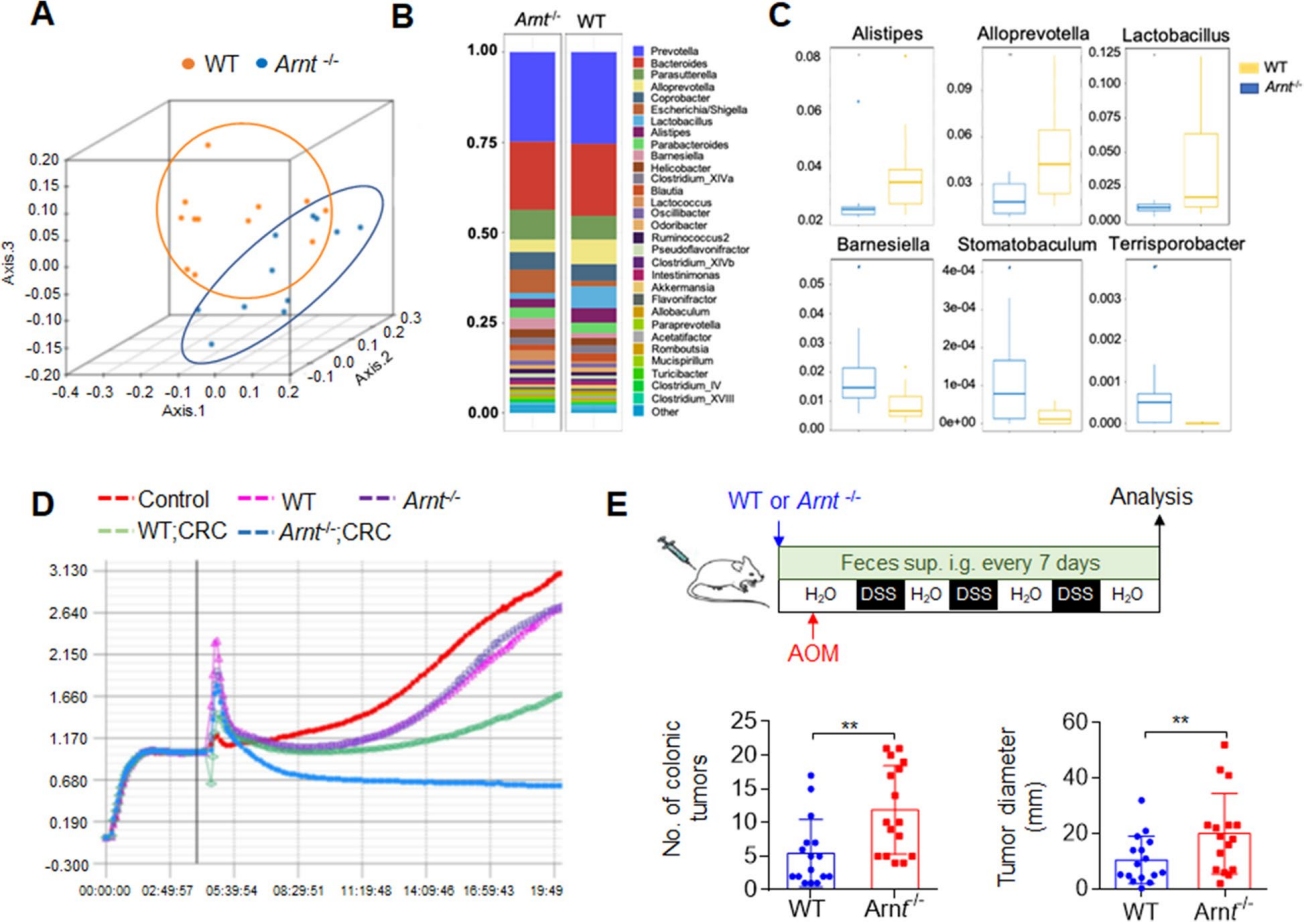
Neutrophil ***Arnt***^−/−^alters the gut microbiota in mice with colorectal cancer. Analysis of feces and fecal metabolites in WT and *Arnt*^−/−^ mice. Feces were collected at the end of the experiment. Half of the fecal samples were used for DNA isolation and sequencing, and the other half were used for stimulating HT-29 cells. **A** Unweighted UniFrac-based PCoA plot based on all OTUs. **B** Total OTU sequences taxonomically assigned to bacterial genera. **C** Significantly different bacteria at the genus level between the WT and *Arnt*^−/−^ groups. The graph summarizes data from three independent experiments with eleven or twelve mice per group (**A**-**C**). **D** RCTA indicated the effect of fecal metabolites on intestinal epithelial cells. Metabolites from cancer-induced mice obviously affected HT-29 cell adhesion and growth (green line and blue line), especially metabolites from *Arnt*^−/−^ mice, which caused most HT-29 cells to die (blue line). Representative of three independent experiments. **E** The feces of WT and *Arnt*^−/−^ mice were collected and intragastrically administered to WT mice every other day for induction of colorectal cancer by azoxymethane (AOM) injection and the addition of dextran sulfate sodium salt (DSS) to the drinking water for 80 days. The number of colonic tumors (left) and the tumor diameter (right) are summarized. The graph summarizes data from four independent experiments with sixteen mice per group. **, *P* < 0.01, compared with the indicated groups

The microbiota has been shown to play an important role in the occurrence and development of colorectal cancer, but the metabolic changes of the microbiota in immune regulation, especially in neutrophil immune regulation, have not been clear. Therefore, we observed the effects of ARNT deletion of neutrophils on the microbiota in mice with colorectal cancer. We analyzed the feces and fecal metabolites in WT and *Arnt*^−/−^ mice with colorectal cancer. Feces were collected at the end of the experiment and used for DNA isolation and sequencing. We found that the composition of the gut microbiota in the *Arnt*^−/−^ mice was different from that in the WT control group (Fig. 5A-B). Among these different bacteria, *Alistipes, Alloprevorwlla* and *Lactobacillus* were clearly more prevalent in the WT group, but *Barnesiella, Stomatobaculun and Terrisporobacter* were less prevalent in the WT group (Fig. 5C), which indicated that these microbes may be related to colorectal cancer development. In addition to bacteria, metabolites from gut microbiota are also important influencers regulating the host response. RTCA was used to detect the effect of metabolites on HT-29 cells. The results showed that fecal supernatant from cancer mice (green and blue lines) significantly affected cell growth compared with that of normal mice (pink and purple lines) (Fig. 5D). In particular, metabolites from *Arnt*^−/−^ mice caused most HT-29 cells to die (Fig. 5D), and their ability to stimulate cytokines in RAW264 cells was lower than that of WT mice (Fig. S9A-B). These data suggest that gut microbiota and metabolite alterations are probably involved in *Arnt*^−/−^ neutrophil functional regulation against colorectal cancer.

To further evaluate the regulatory effect of ARNT-deficient neutrophils on bacterial flora changes, feces from colorectal cancer WT and *Arnt*^−/−^ mice were collected. Two kinds of fecal supernatant were given to normal WT mice by gavage during the induction of colorectal cancer, and the numbers and diameters of tumors were detected. The results showed that the feces of neutrophils *Arnt*^−/−^ mice could still significantly promote tumor incidence and growth (Fig. 5E). These results indicate that stool could replicate the colorectal cancer effects of neutrophils *Arnt*^−/−^ mice. Additionally, *Arnt*^−/−^ neutrophils may alter the gut microbiota of mice, and alterations in the gut microbiota composition and metabolites are the main reasons for the development of colorectal cancer induced by *Arnt*^−/−^ neutrophils.

### ***Arnt***^−/−^neutrophils promoted colorectal cancer growth by altering the gut microbiota

**Fig. 6:**
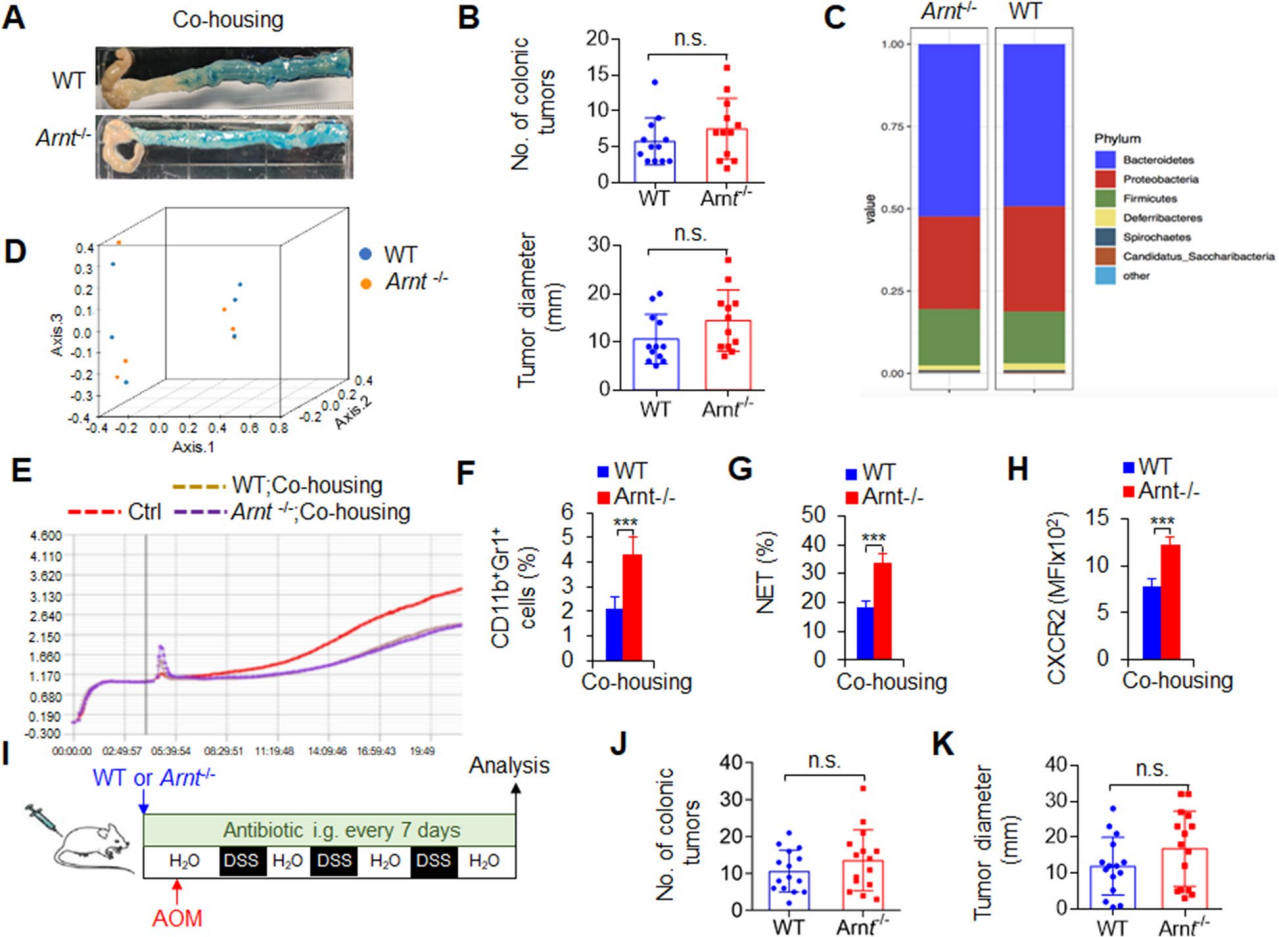
Neutrophil ***Arnt***^−/−^promoted colorectal cancer growth by altering the gut microbiota. WT and *Arnt*^−/−^ mice were cohoused and underwent colorectal cancer induction for 80 days after the injection of azoxymethane (AOM) and the addition of dextran sulfate sodium salt (DSS) to the drinking water. The number of colonic tumors (**A**) and the tumor diameter (**B**) are summarized. Representative of three independent experiments (**A**). The graph summarizes data from three independent experiments with twelve mice per group (**B**). (**C-D**) Analysis of feces and fecal metabolites in WT and *Arnt*^−/−^ mice. Feces were collected at the end of the experiment. Feces were used for DNA isolation and sequencing. (**C**) Total OTU sequences taxonomically assigned to bacterial genera. Each bar indicates (**D**) an unweighted UniFrac-based PCoA plot based on all OTUs and represents the mean of the microbiota composition. Representative of two independent experiments with five or six mice per group. (**E**) Analysis of feces and fecal metabolites in WT and *Arnt*^−/−^ mice. Feces were collected at the end of the experiment and used to stimulate HT-29 cells. Metabolites from cancer-induced mice obviously affected HT-29 cell adhesion and growth (green and blue lines), especially metabolites from *Arnt*^−/−^ mice, which caused most HT-29 cells to die (blue line). Representative of three independent experiments. (**F**) Percentage of tumor-infiltrating neutrophils in the colon. (**G**) Tumor-infiltrating neutrophils were isolated and stimulated with LPS in vitro as indicated to detect NETs. The percentage of NETs-forming cells was quantified. (**H**) MFI of CXCR2 in tumor-infiltrating neutrophils. Representative of three independent experiments with four mice per group (**F**-**H**). (**I-K**) Colorectal cancer induction for 80 days after the injection of AOM and the addition of DSS to the drinking water and weekly antibiotic injection, as indicated in **I**. The number of colonic tumors (**J**) and the tumor diameter (**K**) are summarized. The graph summarizes data from three independent experiments with fifteen mice per group (**J**-**K**). ***P < 0.001, compared with the indicated groups. n.s., not significant

Coprophagy is widespread among mice, which indicates a spontaneous exchange of gut microbiota. Thus, cohousing WT and *Arnt*^−/−^ mice is a noninvasive method to evaluate the role of gut microbiota and their metabolites. To further clarify the role of neutrophils *Arnt*^−/−^ in promoting the occurrence and development of colorectal cancer, we cohoused WT and *Arnt*^−/−^ mice to exclude the influence of changes in the gut microbiota. The results showed that the differences in tumor incidence and tumor growth between WT and *Arnt*^−/−^ mice disappeared after cohousing (Fig. 6A-B). Feces were collected at the end of the experiment and used for DNA isolation and sequencing. We found that cohousing eliminated the difference in the gut microbiota between the two groups (Fig. 6C-D). Furthermore, the different effects of fecal metabolites on intestinal epithelial HT-29 cell adhesion and growth and the effects of cytokine production on RAW264.7 cells, were comparable in WT and *Arnt*^−/−^ mice (Figs. 6E and S10A-B). These data suggest that gut microbiota or their metabolites are sufficient for colorectal cancer development induced by *Arnt*^−/−^ neutrophils. However, *Arnt*^−/−^ mice still showed a higher percentage of CD11b^+^Gr1^+^ neutrophils, more NET formation and higher CXCR2 expression in neutrophils than WT mice, even though these mice were cohoused (Fig. 6F-H). These data collectively suggest that gut microbiota regulation is required for colorectal cancer development downstream of neutrophil functional regulation induced by *Arnt*^−/−^ in mice.

Interestingly, if the mice were treated weekly with antibiotics during the induction of colorectal cancer, the gut microbiota of mice could be effectively eliminated, and the effect of *Arnt*^−/−^ mice on the development of colorectal cancer could be significantly restored (Fig. 6I-K). These data suggest that gut microbiota or their metabolites are necessary for colorectal cancer induced by *Arnt*^−/−^ neutrophils. Thus, *Arnt*^−/−^ neutrophils enhanced the recruitment and function of neutrophils in a CXCR2-dependent manner coupled with gut microbiota in developing colorectal cancer in mice.

### Pharmacological targeting of ARNT modulates neutrophils coupled with the gut microbiota in colorectal cancer

**Fig. 7:**
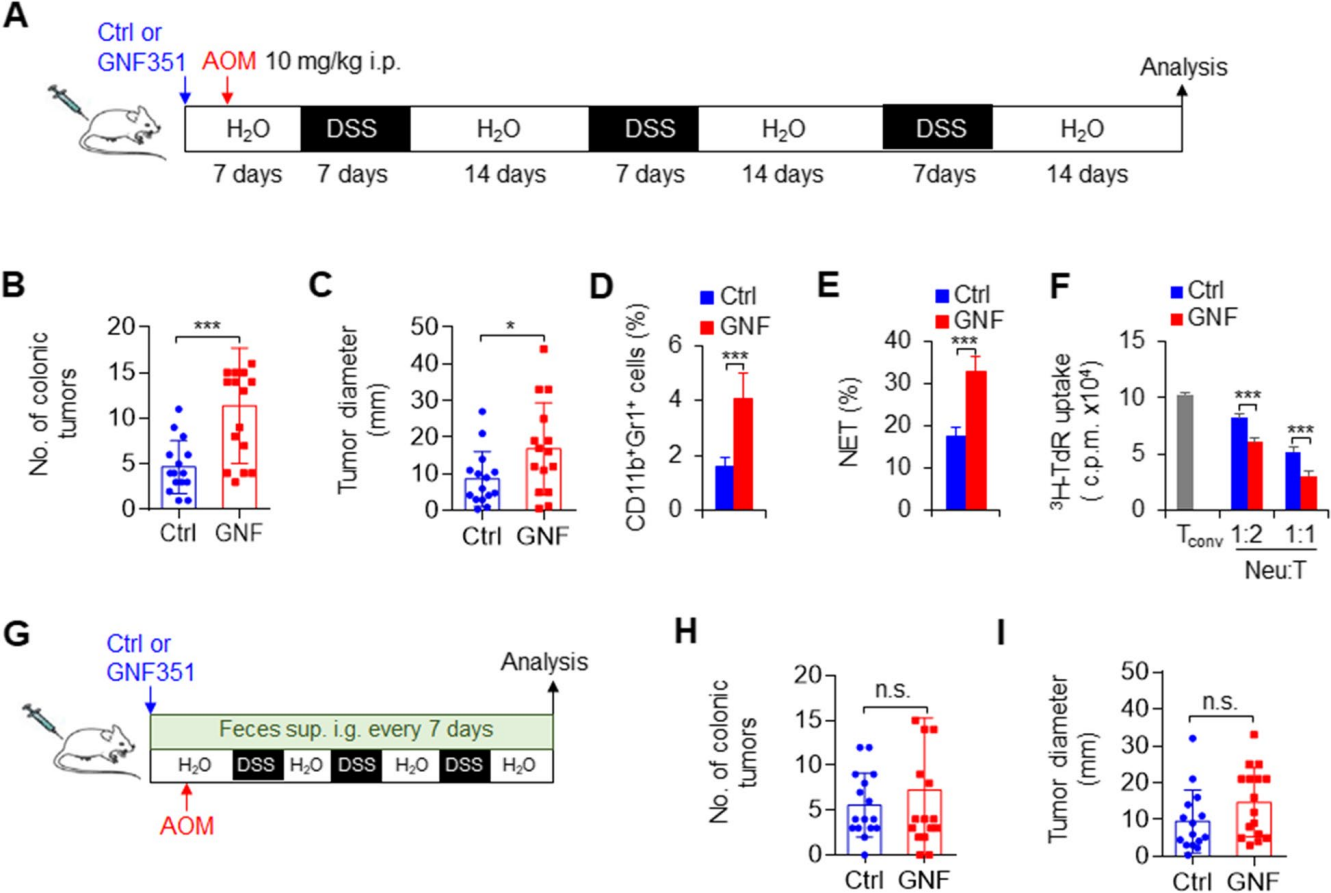
ARNT drives neutrophil recruitment, function and the gut microbiota in anti-colorectal cancer treatment. **A-F** Mouse colorectal cancer induction for 80 days after the injection of azoxymethane (AOM) and the addition of dextran sulfate sodium salt (DSS) to the drinking water. Meanwhile, an ARNT inhibitor (GNF351, 5 mg/kg body weight) was i.p. injected weekly as indicated in **A**. The number of colonic tumors (**B**) and the tumor diameter (**C**) are summarized. The graph summarizes data from three independent experiments with fifteen mice per group (**B**-**C**). **D** Percentage of tumor-infiltrating neutrophils in the colon. **E** Tumor-infiltrating neutrophils were isolated and stimulated with LPS in vitro as indicated to detect NETs. The percentage of NETs-forming cells was quantified. **F** Suppressive assay of tumor-infiltrating neutrophils from tumor-bearing mice. Data from coculture with allogeneic CD4^+^ T cells with different ratio between neutrophils and T cells are shown. Representative of three independent experiments with three mice per group (**D**-**F**). **G-I** Mouse colorectal cancer induction for 80 days after the injection of AOM and the addition of DSS to the drinking water. Meanwhile, GNF351 (5 mg/kg body weight) was i.p. injected weekly, and the feces of WT mice were intragastrically administered to these mice every other day as indicated in **G**. The number of colonic tumors (**H**) and the tumor diameter (**I**) are summarized. The graph summarizes data from three independent experiments with sixteen mice (**H-I**). *, *P* < 0.05 and ***P < 0.001, compared with the indicated groups. n.s., not significant

Next, we sought to apply a pharmacological approach to target ARNT in neutrophils and determine its effects on neutrophil function and colorectal cancer development to test whether we can recapitulate our findings from genetically targeting ARNT. We applied GNF351, an effective inhibitor of the ARNT signaling pathway, to mice by inhibiting the AHR upstream of ARNT, as indicated in Fig. 7A. Consistent with the results following the genetic depletion of ARNT, we observed that inhibiting ARNT significantly enhanced the incidence and growth of colorectal cancer (Fig. 7B-C) and increased the percentage of CD11b^+^Gr1^+^ cells (Fig. 7D), NET formation (Fig. 7E) and suppressive activities (Fig. 7F) of neutrophils compared with those of the control groups. Interestingly, in the process of inducing colorectal cancer, in addition to GNF351 treatment, the feces of normal WT colorectal cancer mice were administered by gavage once a week for intervention treatment. We observed that although GNF351 treatment could still cause a higher incidence and growth of colorectal cancer (Fig. 7B-C), these effects could be recovered by gavage of the feces of normal WT mice (Fig. 7G-I). Thus, these data suggest that ARNT-mediated neutrophil recruitment and function combined with gut microbiota regulation probably contribute to colorectal cancer development in mice.

Next, we applied a pharmacological approach to target ARNT in human neutrophils and determine whether we can recapitulate our finding in genetic targeting ARNT. ARNT expressions were determined in human neutrophils, which is from human peripheral blood (Fig. S11A). We applied GNF351, an effective inhibitor of the ARNT signaling pathway, to human neutrophils by inhibiting the AHR upstream of ARNT. The pharmacological inhibition of ARNT in human neutrophils largely recapitulated what we observed in genetic mouse neutrophils in terms of the production of TNFα and IL-10 and expression of CXCR2 in human neutrophils (Fig. S11A-C). Thus, our data demonstrated that ARNT mediated an evolutionary conserved signaling pathway in both mouse and human neutrophils.

## Discussion

In the present study, we established that an underappreciated function of the transcription factor ARNT in colorectal cancer is to limit the recruitment and function of neutrophils coupled with the gut microbiota to enable colitis-associated colorectal cancer progression (Fig. S12). ARNT deficiency enhances the migration and functional activities of neutrophils under physiological conditions and pathological tumor microenvironment. Although ARNT plays an important role in regulating the function and recruitment of tumor-infiltrating neutrophils, it is obvious that these effects depend on alterations in gut microbiota homeostasis in the tumorigenic microenvironment. Therefore, these data collectively display the essential relationship between the recruitment and function of neutrophils and homeostasis of the gut microbiota with implications for the combination of immune and gut microbiota regulation as a therapeutic approach for colorectal cancer.

Colorectal cancer includes hereditary, sporadic and colitis-associated colorectal cancers [1, 3, 29]. A large amount of epidemiological and experimental evidence strongly supports the view that chronic inflammation leads to the occurrence, development and metastasis of colorectal cancer [5, 26, 30]. Ulcerative colitis is the most common form of inflammatory bowel disease (IBD) and is associated with an increased risk of colorectal cancer. Because chronic inflammation is related to immunosuppression, a reasonable explanation for the development of chronic inflammation into cancer is a tumor microenvironment induced by immunosuppression and immune tolerance to tumor cells[5, 10]. Neutrophils are one of the key regulatory factors in immune activities [5, 10, 30]. Although the role and mechanism of neutrophils in tumors have been widely studied, the precise effects of ARNT on neutrophils remain unclear. Herein, our data showed that *Arnt*^−/−^ mice significantly promoted local accumulation of neutrophils in tumors, NET formation and secretion of anti-inflammatory cytokines by tumor-infiltrating neutrophils, all of which contribute to the formation of an immune-tolerant tumor microenvironment and promote the occurrence and development of colorectal cancer. Importantly, CD11b^+^Gr1^+^ neutrophils depletion significantly reversed the tumor-promoting effects of *Arnt*^−/−^ mice, suggesting that the absence of ARNT in neutrophils plays a key role in tumorigenesis. CXCR2 is critical for inducing the recruitment and NET formation of neutrophils in cancer [9, 31]. We further investigated the expression of CXCR2 and found that the absence of ARNT significantly promoted the expression of CXCR2 in neutrophils under tumor or physiological conditions. However, blocking or inhibiting CXCR2 expression significantly downregulated local neutrophil accumulation, NET formation and proinflammatory cytokine production. These results collectively suggest that CXCR2 is required for the regulatory effect of ARNT on neutrophil recruitment and suppressive activities. Additionally, these data also suggest that ARNT is important for all neutrophil populations with regulatory activities under physiological or tumor conditions. However, the present phenotypic analysis cannot distinguish different neutrophils’ populations, and neutrophil origin cells should also be included in CD11b^+^Gr1^+^ neutrophils, such as myeloid-derived suppressor cells (MDSCs) from neutrophils origin.

ARNT is a transcription factor and plays a key role in signaling processes involving AHR and HIF [12, 32]. AHR is a cytosolic bHLH PAS transcription factor that consists of domains similar to ARNT [33]. Appropriate compounds are bound in the PAS domain of AHR, which leads to conformational changes and reveals a complete domain [34]. AHR is then transferred to the nucleus, where it is immortalized with ARNT. The transcriptionally active AHR/ARNT complex binds to exogenous response elements in the regulatory region of the target gene and initiates transcription [35]. AHR has been shown to play an important role in neutrophils induction and mobilization [36, 37], but the precise role of ARNT in neutrophils remains unclear. It is generally believed that its expression is constant and may not play an important role. However, recent studies suggest that ARNT may also play an important role, especially in tumor growth [38]. One study overexpressed ARNT in Hepa-1 cells in vitro, and according to the kinetic data, ARNT probably plays an important role in the early stage of tumor growth. In addition, the authors suggested that ARNT as a drug target is superior to HIF1α expression in some tumors of the control group [32, 38, 39]. A recent study confirmed the role of ARNT as a potential target in the treatment of cancer, which linked the expression of the bHLH PAS transcription factor with cisplatin resistance [40]. Cisplatin resistance is mediated by the ARNT/SP1-dependent transcription of the multidrug resistance 1 gene, which encodes an ATP-binding protein cassette transporter that promotes the action of this anticancer drug [40]. Herein, our data demonstrated that ARNT negatively regulated the recruitment and function of neutrophils under physiological conditions or in the tumor microenvironment. ARNT deficiency displayed similar effects to AHR deficiency in neutrophils which is based most likely on a disrupted canonical AHR signaling pathway by the lack of its dimerization partner ARNT.

Recently, a large number of studies have shown that changes in the gut microbiota are key regulatory factors in the occurrence and development of colorectal cancer [4]. Using 16 S rRNA sequencing of the gut microbiome, we noted that MDSC *Arnt*^−/−^ mice exhibited alterations in the gut microbiome and metabolome in colorectal cancer. It was found that the feces of ARNT-deficient mice could also replicate the tumor-promoting effect of ARNT-deficient neutrophils. Cohousing WT and *Arnt*^−/−^ mice to eliminate ARNT-deficient mice with different flora or antibiotics to eliminate the flora significantly weakened the protumor effect of ARNT-deficient neutrophils. These results suggest that the functional changes in neutrophils induced by ARNT deficiency could promote the occurrence and development of tumors by changing the gut microbiota. These results also provide experimental evidence for the future combination of immune and microbiological treatment studies of colitis-associated colorectal cancer.

## Conclusion

In summary, we found that ARNT governs the recruitment and functional activities of regulatory neutrophils and modulates the alteration of the gut microbiota in colitis-associated colorectal cancer pathological microenvironments. ARNT deficiency displays similar effects to AHR deficiency in neutrophils. CXCR2 signaling is required for the recruitment and functional regulation of neutrophil activities induced by ARNT, which conferred protection against colorectal tumorigenesis. Our results defined a new role for the transcription factor ARNT in regulating neutrophils recruitment and function and the gut microbiota with implications for the future combination of gut microbiota and immunotherapy approaches in colorectal cancer.

## Supplementary Information

**Additional file 1: Supplementary Table 1.** Primer sequences used for real-time PCR assays.

**Additional file 2: Fig. S1.** *Arnt*^−/−^ enhanced the migration and function of CD11b^+^Gr1^+^ neutrophils. (A) Gating strategy of viability dye to exclude dead cells (7-ADD^+^ cells) to stain CD11b^+^Gr1^+^ neutrophils in BMs in C57BL/6 mice with flow cytometry. (B-D) The colon of mice was isolated and photographed (B) and hematoxylin-eosin staining of colon (C). (D) Flow cytometry of the mean fluorescence intensity (MFI) of CXCR2 expression in splenic CD11b^+^Gr1^+^ cells from WT and *Arnt* mice. Data are representative of three independent experiments with three mice per group. ***P <0.001, compared with the indicated groups. **Fig. S2.** Arnt^-/-^ enhanced the migration and functions of CD11b^+^Gr1^+^ neutrophils. (A-E) BM cells stimulated with GM-CSF for 4 days to induce CD11b^+^Gr1^+^ neutrophils in vitro. Percent of CD11b^+^Gr1^+^ neutrophils (A), percent of NET-forming cells in CD11b^+^Gr1^+^ neutrophils (B), percent of intracellular staining of IL-10 in CD11b^+^Gr1^+^ neutrophils (C) and MFI of CXCR2 in CD11b^+^Gr1^+^ neutrophils (D). Production of indicated chemokines in supernatant with ELISA (E). (F-I) BM cells stimulated with GM-CSF for 4 days to induce CD11b^+^Gr1^+^ neutrophils in vitro. Sorted CD11b^+^Gr1^+^ cells were transfected with control shRNA or AHR shRNA vector. AHR mRNA expression of CD11b^+^Gr1^+^ cells (F), percent of NET-forming cells in CD11b^+^Gr1^+^ neutrophils (G), percent of intracellular staining of IL-10 (H) and MFI of CXCR2 expression (I) in CD11b^+^Gr1^+^ neutrophils. Data are representative of three independent experiments with three mice per group. **P <0.01 and ***P <0.001, compared with the indicated groups. **Fig. S3.** ARNT inhibits the functions of CD11b^+^Gr1^+^ neutrophils. BM cells stimulated with GM-CSF for 4 days to induce CD11b^+^Gr1^+^ neutrophils in vitro. Sorted CD11b^+^Gr1^+^ cells were transfected with control retrovirus (RV) or ARNT-expressing retrovirus (ARNT-RV). (A) Percent of NET-forming cells in GFP^+^CD11b^+^Gr1^+^ cells transduced with RV or ARNT-RV. (B-C) Intracellular staining of TNFα (C) and IL-10 (D) in GFP^+^CD11b^+^Gr1^+^ cells transduced with RV or ARNT-RV. (D) Suppressive activity assay of sorted GFP^+^CD11b^+^Gr1^+^ cells transduced with RV or ARNT-RV. Data are representative of three independent experiments with three mice per group. *P <0.05 and ***P <0.001, compared with the indicated groups. **Fig. S4.** *Arnt*^-/-^ did not alter the expression of AHR signaling molecules in neutrophils. Western blot of indicate molecular expression in CD11b^+^Gr1^+^ neutrophils induced by GM-CSF (10 ng/ml) for 4 days from BMs of WT and *Arnt*^-/-^ mice. Data are representative of three independent experiments. **Fig. S5.** *Arnt*^-/-^ tumor microenvironment enhances CD11b^+^Gr1^+^ neutrophil recruitment and functions in promoting the colorectal cancer growth. Colorectal cancer induction for 80 days after the injection of azoxymethane (AOM) and dextran sulfate sodium salt (DSS) drinking water. (A) Changes of body weight. Graph summarizes data from three independent experiments with thirteen mice per group. (B) Percent of CD11b^+^Gr1^+^ neutrophils, CD11b^+^Gr1^+^Ly6C^hi^CD115^+^ cells and CD11b^+^Gr1^+^Ly6G^hi^CD115^-^ cells in BMs, Blood and Spleen. Dot-plots present the representative data from flow cytometry analysis is shown in left and data shown in right. Representative of three independent experiments with three mice per group. (C) Tumor-infiltrating CD11b^+^Gr1^+^ neutrophils were isolated and stimulated with LPS in vitro as indicated to detect the NET. The area of NET is quantified. Representative of three independent experiments with three mice per group. **P <0.05 and ***P <0.001, compared with the indicated groups. **Fig. S6.** *Arnt*^-/-^ tumor microenvironment enhances CD11b^+^Gr1^+^ neutrophil recruitment and functions in promoting the colorectal cancer growth. Colorectal cancer induction for 80 days after the injection of azoxymethane (AOM) and dextran sulfate sodium salt (DSS) drinking water. (A-B) Intracellular staining of TNFα in CD11b^+^Gr1^+^ neutrophils from colon and MLN (A) and BMs, blood and spleen (B). Dot-plots present the representative data from flow cytometry analysis is shown in left and data summary shown in right. (C) Indicated chemokine mRNA expression of tumor-infiltrating CD11b^+^Gr1^+^ neutrophils from WT and *Arnt*^-/-^ mice. (D) MFI of CXCR2 in CD11b^+^Gr1 ^+^ neutrophils from colon and MLN of WT and *Arnt*^-/-^ mice. (E) Indicated gene mRNA expression of tumor-infiltrating CD11b^+^Gr1^+^ neutrophils from WT and *Arnt*^-/-^ mice. Data are representative of three independent experiments with at least three mice per group. ***P <0.001, compared with the indicated groups. **Fig. S7.** *Arnt*^-/-^ tumor microenvironment enhances CD11b^+^Gr1^+^ neutrophil recruitment and functions in promoting the colon cancer growth. (A-B) MC38 tumor cells were implanted subcutaneously in WT and *Arnt*^-/-^ mice (*n*=10) and tumor growth size was measured every 3 days for 18 days. The representative tumor photo (A) is shown and tumor growth curve (B) are summarized. (C) Percent of CD11b^+^Gr1^+^ neutrophils in tumor and dLN of WT and *Arnt*^-/-^ tumor-bearing mice at day 18. (D-E) Intracellular staining of TNFα (D) and IL-10 (E) expression in CD11b^+^Gr1^+^ cells of tumor and dLN from WT and *Arnt*^-/-^ mice at day 18. Dot-plots present the representative data from flow cytometry analysis is shown on the left, and the data are summarized on the right. (F) MFI of CXCR2 in CD11b^+^Gr1^+^ neutrophils of tumor and dLN from WT and *Arnt*^-/-^ mice by flow cytometry. Representative of three independent experiments with three to four mice per group. **P <0.01 and ***P <0.001, compared with the indicated groups**. Fig. S8.** Tumor microenvironment enhances *Arnt*^-/-^ CD11b^+^Gr1^+^ neutrophil recruitment and functions in promoting the colorectal cancer growth. 5 × 10^6^ BM neutrophils (Neu.) were isolated from WT or *Arnt*^-/-^ mice every 7 days and transferred to WT recipient mice twice by i.v. injection. The recipient WT mice were injected with azomethane (AOM) and dextran sulfate sodium salt (DSS) drinking water to induce colorectal cancer. (A) Carton of experimental program. (B) Summary of No. of colonic tumor. (C) Summary of tumor diameter. Graph summarizes the data from three independent experiments with fifteen mice per group. *, *P* <0.05 and ***P <0.001, compared with the indicated groups. **Fig. S9.** Analysis fecal metabolites in WT and *Arnt*^-/-^ colorectal cancer mice. Fecal supernatant was used to stimulate RAW264.7 cell for 3 hrs, and cell culture supernatant were collected and the IL-1β (A) and TNFα (B) levels were detected in culture supernatant by ELISA. Data are representative of three independent experiments with at least three mice per group. *, *P* <0.05 and ***P <0.001, compared with the indicated groups. **Fig. S10.** Analysis fecal metabolites in co-housed WT and *Arnt*^-/-^ mice. Fecal supernatant was used to stimulate RAW264.7 cell for 3 hrs, and the culture supernatants were collected and the IL-1β (A) and TNFα (B) levels were detected in culture supernatant by ELISA. Representative of three independent experiments with three mice per group. n.s., not significant. **Fig. S11.** ARNT regulates the functions of neutrophils in human. Human neutrophils treated by GNF351 in vitro for 12 hrs. (A) mRNA expression of ARNT in neutrophils treated by GNF351 (500 nM). (B) Level of TNFα and IL-10 concentration in culture supernatant by ELISA. (C) Expression of CXCR2 in neutrophils by flow cytometry. Data are representative of three independent experiments (*n*=3-4). *, *P*<0.05 and ***P<0.001, compared with the indicated groups. **Fig. S12.** ARNT limits the recruitment and function of neutrophils in colorectal cancer by regulating gut microbiota. Under inflammatory stimulus, ARNT deletion of CD11b^+^Gr1^+^ neutrophils can significantly up-regulate CXCR2 expression, promote CD11b^+^Gr1^+^ neutrophil recruitment and NET formation, further trigger changes in gut microbiota and metabolites, and play an important role in the anti-colitis-associated colorectal cancer in tumor microenvironment (TME).

## Data Availability

All data generated or analyzed during this study are included in this published article and its supplementary information files.

## Abbreviations

AHR: Aryl hydrocarbon receptor
ARNT: Aryl hydrocarbon receptor nuclear translocator
BM: Bone marrow
CMPs: Common myeloid progenitors
GI: Gastrointestinal
HSCs: Hematopoietic stem cells
HIF: Hypoxia inducible factor
IHC: Immunohistochemistry
IBD: Inflammatory bowel disease
MPPs: Multipotent progenitors
GMPs: Granulocyte-monocyte progenitors
MDSCs: Myeloid-derived suppressor cells
NET: Neutrophil extracellular trap
RBCs: Red blood cells
RTCA: Real-time cellular analysis, TANs:Tumor-associated neutrophils.
