# Supplementary material for: Aryl hydrocarbon receptor nuclear translocator limits the recruitment and function of regulatory neutrophils against colorectal cancer by regulating the gut microbiota
Source: J Exp Clin Cancer Res. 2023 Mar 1;42:53. doi: 10.1186/s13046-023-02627-y (PMC9976387; doi:10.1186/s13046-023-02627-y)
Supplement: Supplementary file 1 — Additional file 1: Supplementary Table 1. Primer sequences used for real-time PCR assays. [file 13046_2023_2627_MOESM1_ESM.pdf]

Supplementary Table.1 Primer sequences used for real-time PCR assays

	Forward primer	Reverse primer
Arnt	5'-TCTCCCTCCCAGATGATGAC-3’	5'-CAATGTTGTGTCGGGAGATG-3’
Ahr	5'-CTGGTTGTCACAGCAGATGCCT-3'	5'-CGGTCTTCTGTATGGATGAGCTC-3'
Ccl3	5'-ACTGCCTGCTGCTTCTCCTACA-3'	5'-ATGACACCTGGCTGGGAGCAAA-3'
Ccl5	5'-CCTGCTGCTTTGCCTACCTCTC-3'	5'-ACACACTTGGCGGTTCCTTCGA-3'
Cxcl1	5'-GCACCCAAACCGAAGTCATAG-3'	5'-AGAAGCCAGCGTTCACCAGA-3'
Cxcl2	5'-GCCCAGACAGAAGTCATAGCC-3'	5'-CTCCTCCTTTCCAGGTCAGTTA-3'
Ahrr	5'-ACATACGCCGGTAGGAAGAGA-3'	5'-GGTCCAGCTCTGTATTGAGGC-3'
Cyp1a1	5'-CATCACAGACAGCCTCATTGAGC-3'	5'-CTCCACGAGATAGCAGTTGTGAC-3'
Cyp1a2	5′-CATCCCCCACAGCACAACGA-3′	5′-GGTAAGAAACCGCTCTGGGC-3′
Cyp1b1	5'-GCCACTATTACGGACATCTTCGG-3'	5'-ACAACCTGGTCCAACTCAGCCT-3'
Cox2	5'-GCGACATACTCAAGCAGGAGCA-3'	5'-AGTGGTAACCGCTCAGGTGTTG-3'
Hprt	5'-CTGGTGAAAAGGACCTCTCGAAG-3'	5'-CCAGTTTCACTAATGACACAAACG-3'
