# Supplementary material for: Aryl hydrocarbon receptor nuclear translocator limits the recruitment and function of regulatory neutrophils against colorectal cancer by regulating the gut microbiota
Source: J Exp Clin Cancer Res. 2023 Mar 1;42:53. doi: 10.1186/s13046-023-02627-y (PMC9976387; doi:10.1186/s13046-023-02627-y)
Supplement: Supplementary file 2 — Additional file 2: Fig. S1. Arnt−/− enhanced the migration and function of CD11b+Gr1+ neutrophils. (A) Gating strategy of viability dye to exclude dead cells (7-ADD+ cells) to stain CD11b+Gr1+ neutrophils in BMs in C57BL/6 mice with flow cytometry. (B-D) The colon of mice was isolated and photographed (B) and hematoxylin-eosin staining of colon (C). (D) Flow cytometry of the mean fluorescence intensity (MFI) of CXCR2 expression in splenic CD11b+Gr1+ cells from WT and Arnt mice. Data are representative of three independent experiments with three mice per group. ***P <0.001, compared with the indicated groups. Fig. S2. Arnt-/- enhanced the migration and functions of CD11b+Gr1+ neutrophils. (A-E) BM cells stimulated with GM-CSF for 4 days to induce CD11b+Gr1+ neutrophils in vitro. Percent of CD11b+Gr1+ neutrophils (A), percent of NET-forming cells in CD11b+Gr1+ neutrophils (B), percent of intracellular staining of IL-10 in CD11b+Gr1+ neutrophils (C) and MFI of CXCR2 in CD11b+Gr1+ neutrophils (D). Production of indicated chemokines in supernatant with ELISA (E). (F-I) BM cells stimulated with GM-CSF for 4 days to induce CD11b+Gr1+ neutrophils in vitro. Sorted CD11b+Gr1+ cells were transfected with control shRNA or AHR shRNA vector. AHR mRNA expression of CD11b+Gr1+ cells (F), percent of NET-forming cells in CD11b+Gr1+ neutrophils (G), percent of intracellular staining of IL-10 (H) and MFI of CXCR2 expression (I) in CD11b+Gr1+ neutrophils. Data are representative of three independent experiments with three mice per group. **P <0.01 and ***P <0.001, compared with the indicated groups. Fig. S3. ARNT inhibits the functions of CD11b+Gr1+ neutrophils. BM cells stimulated with GM-CSF for 4 days to induce CD11b+Gr1+ neutrophils in vitro. Sorted CD11b+Gr1+ cells were transfected with control retrovirus (RV) or ARNT-expressing retrovirus (ARNT-RV). (A) Percent of NET-forming cells in GFP+CD11b+Gr1+ cells transduced with RV or ARNT-RV. (B-C) Intracellular staining of TNFα (C) and [file 13046_2023_2627_MOESM2_ESM.pdf]

Aryl hydrocarbon receptor nuclear translocator limits the recruitment and function of regulatory neutrophils against colorectal cancer by regulating the gut microbiota

Yujing Bi[1, #,*], Qiuli Yang[2,#], Zhengchao Li[1,#], Yuexin Wang[2,#], Yufei Wang[2], Anna Jia[2], Zhiyuan Pan[1], Ruifu Yang[1,a], Guangwei Liu[2,*]

[1]State Key Laboratory of Pathogen and Biosecurity, Beijing Institute of Microbiology and Epidemiology, Beijing 100071 China; [2]Key Laboratory of Cell Proliferation and Regulation Biology, Ministry of Education, College of Life Sciences, Beijing Normal University, Beijing 100875 China;

Supplementary Fig.1

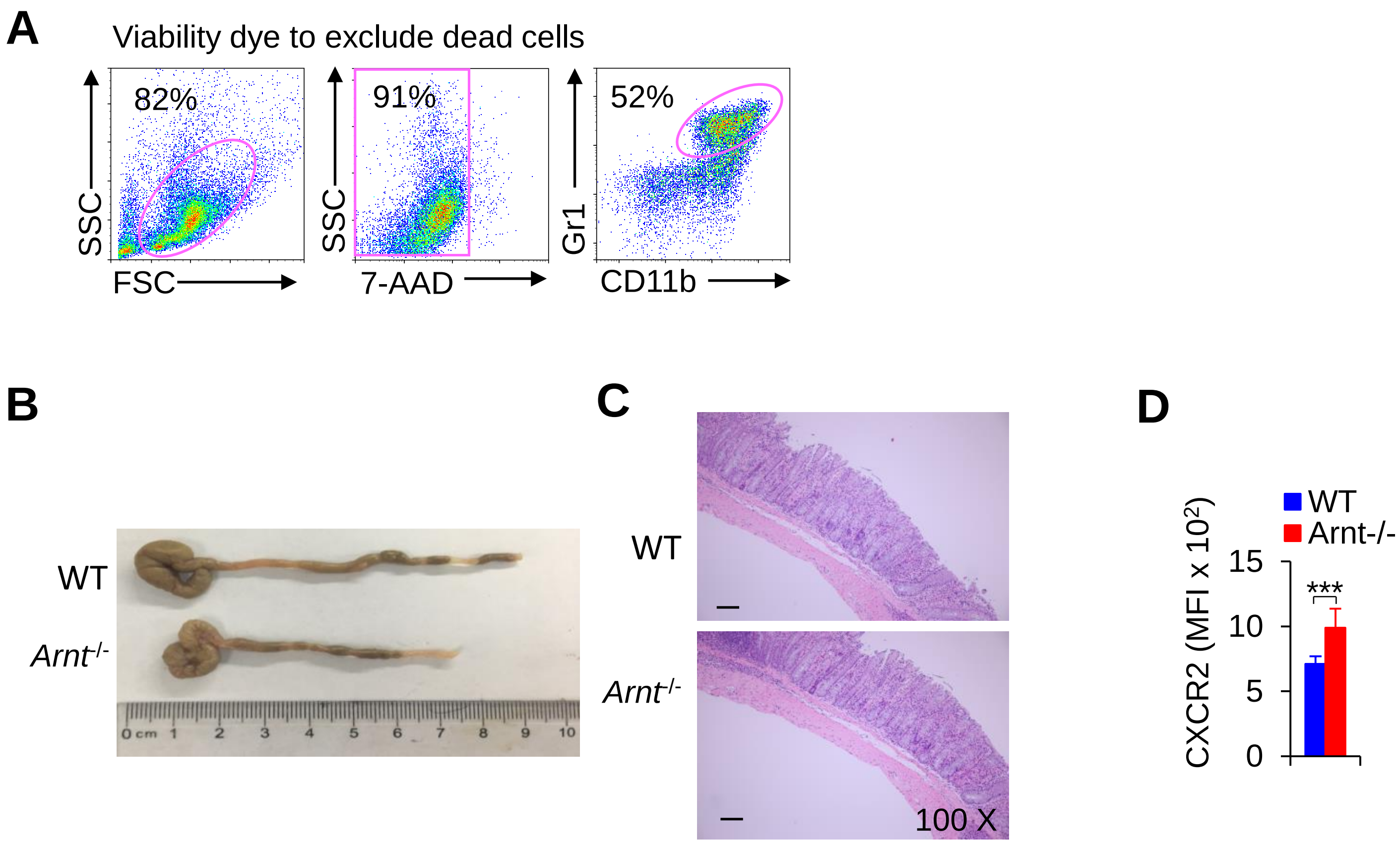

Fig. S1. *Arnt^{-/-}* enhanced the migration and function of CD11b$^+$Gr1$^+$ neutrophils.
(**A**) Gating strategy of viability dye to exclude dead cells (7-ADD$^+$ cells) to stain CD11b$^+$Gr1$^+$ neutrophils in BMs in C57BL/6 mice with flow cytometry. (**B-D**) The colon of mice was isolated and photographed (B) and hematoxylin-eosin staining of colon (C). (D) Flow cytometry of the mean fluorescence intensity (MFI) of CXCR2 expression in splenic CD11b$^+$Gr1$^+$ cells from WT and *Arnt*$^{-/-}$ mice. Data are representative of three independent experiments with three mice per group. ***P <0.001, compared with the indicated groups.

Supplementary Fig.2

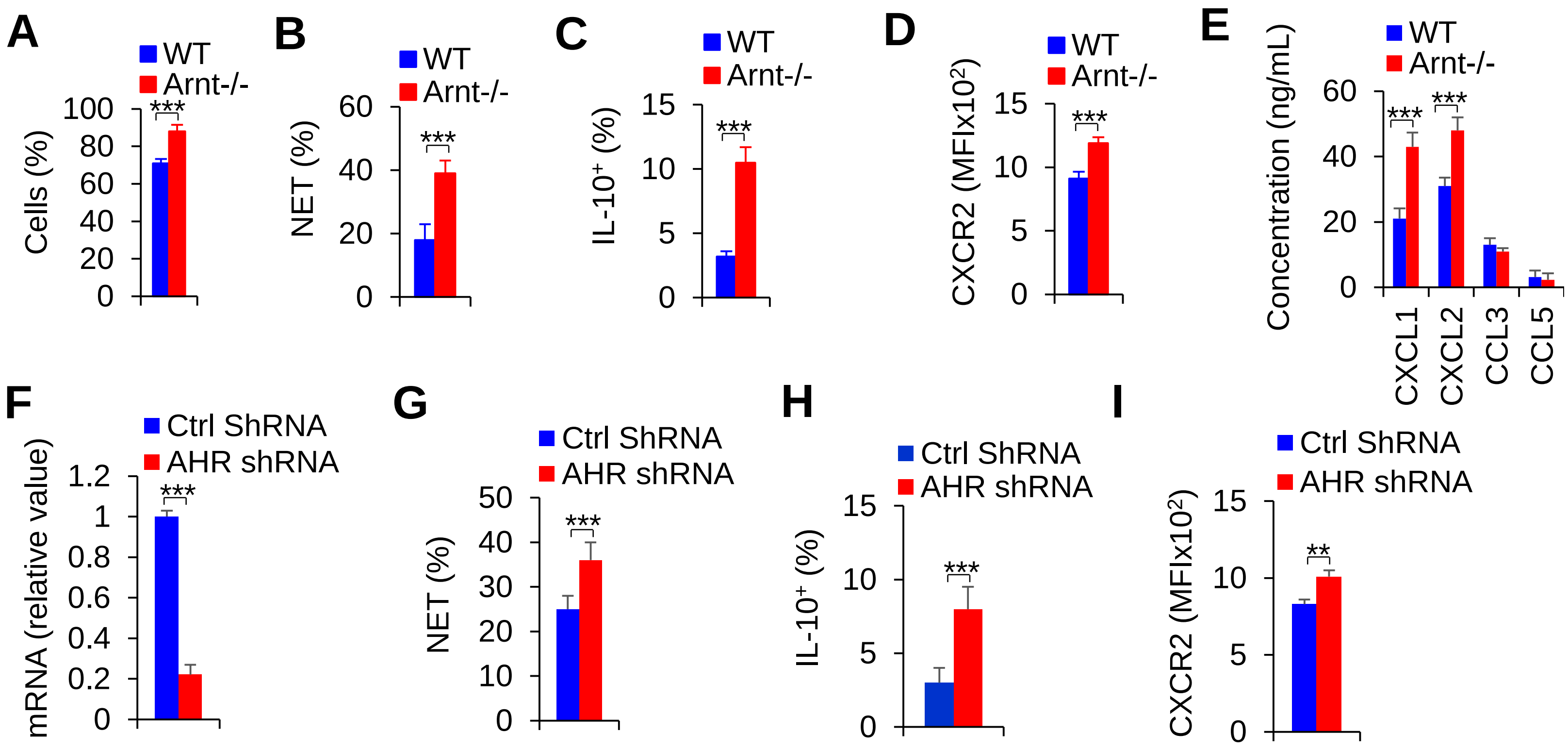

Fig.S2. ***Arnt****$^{-/-}$* **enhanced the migration and functions of $CD11b^+Gr1^+$ neutrophils .**
(**A-E**) BM cells stimulated with GM-CSF for 4 days to induce $CD11b^+Gr1^+$ neutrophils *in vitro*. Percent of $CD11b^+Gr1^+$ neutrophils (A), percent of NET-forming cells in $CD11b^+Gr1^+$ neutrophils (B), percent of intracellular staining of IL-10 in $CD11b^+Gr1^+$ neutrophils (C) and MFI of CXCR2 in $CD11b^+Gr1^+$ neutrophils (D). Production of indicated chemokines in supernatant with ELISA (E). (**F-I**) BM cells stimulated with GM-CSF for 4 days to induce $CD11b^+Gr1^+$ neutrophils *in vitro*. Sorted $CD11b^+Gr1^+$ cells were transfected with control shRNA or AHR shRNA vector. AHR mRNA expression of $CD11b^+Gr1^+$ cells (F), percent of NET-forming cells in $CD11b^+Gr1^+$ neutrophils (G), percent of intracellular staining of IL-10 (H) and MFI of CXCR2 expression (I) in $CD11b^+Gr1^+$ neutrophils. Data are representative of three independent experiments with three mice per group. ***P* <0.01 and ****P* <0.001, compared with the indicated groups.

Supplementary Fig.3

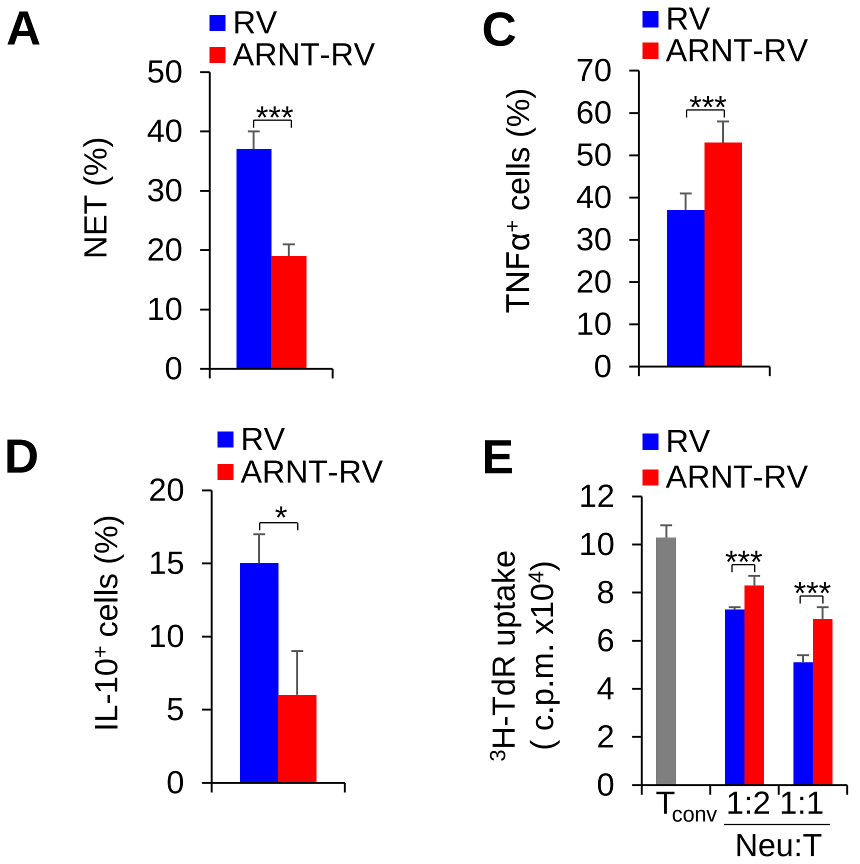

Fig.S3. ARNT inhibits the functions of $CD11b^{+}Gr1^{+}$ neutrophils.

BM cells stimulated with GM-CSF for 4 days to induce $CD11b^{+}Gr1^{+}$ neutrophils in vitro. Sorted $CD11b^{+}Gr1^{+}$ cells were transfected with control retrovirus (RV) or ARNT-expressing retrovirus (ARNT-RV). (**A**) Percent of NET-forming cells in $GFP^{+}CD11b^{+}Gr1^{+}$ cells transduced with RV or ARNT-RV. (**B-C**) Intracellular staining of TNFα (C) and IL-10 (D) in $GFP^{+}CD11b^{+}Gr1^{+}$ cells transduced with RV or ARNT-RV. (**D**) Suppressive activity assay of sorted $GFP^{+}CD11b^{+}Gr1^{+}$ cells transduced with RV or ARNT-RV. Data are representative of three independent experiments with three mice per group. . *P <0.05 and ***P <0.001, compared with the indicated groups.

Supplementary Fig.4

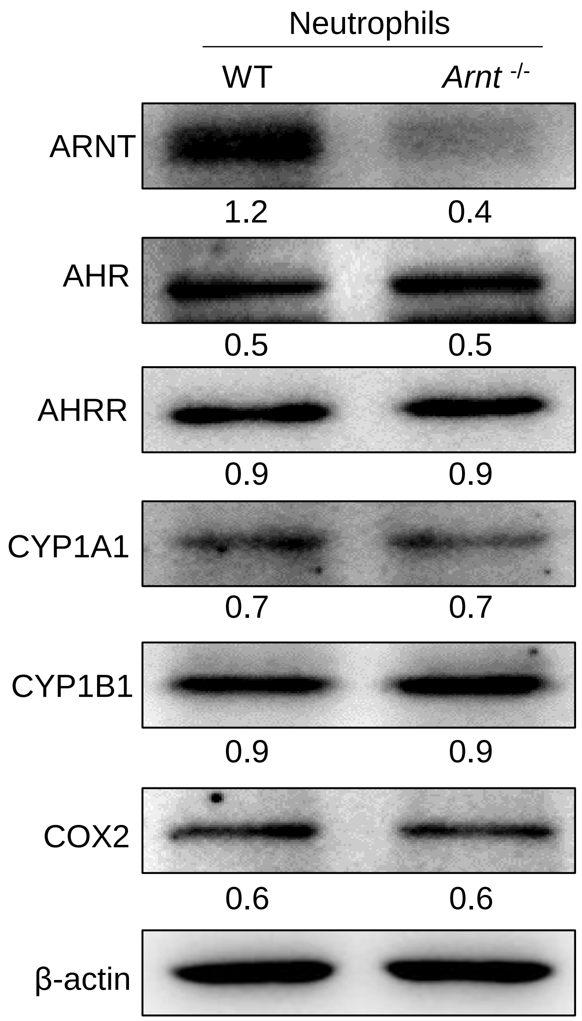

Fig.S4. *Arnt$^{-/-}$* did not alter the expression of AHR signaling molecules in neutrophils.
Western blot of indicate molecular expression in CD11b^{+}Gr1^{+} neutrophils induced by GM-CSF (10 ng/ml) for 4 days from BMs of WT and *Arnt$^{-/-}$* mice. Data are representative of three independent experiments.

Supplementary Fig.5

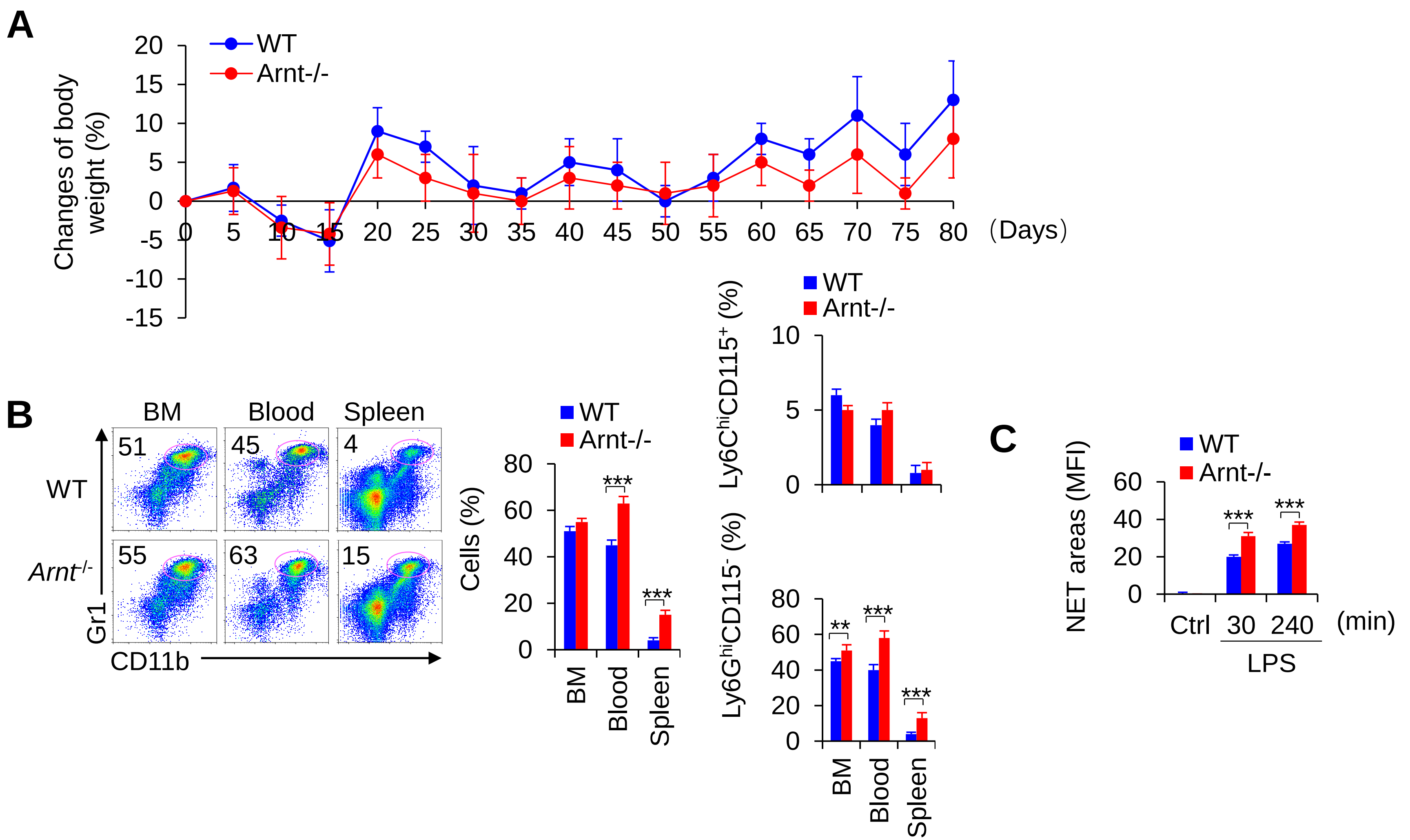

Fig. S5. *Arnt*$^{-/-}$ tumor microenvironment enhances CD11b^{+}Gr1^{+} neutrophil recruitment and functions in promoting the colorectal cancer growth.

Colorectal cancer induction for 80 days after the injection of azoxymethane (AOM) and dextran sulfate sodium salt (DSS) drinking water. (**A**) Changes of body weight. Graph summarizes data from three independent experiments with thirteen mice per group. (**B**) Percent of CD11b^{+}Gr1^{+} neutrophils, CD11b^{+}Gr1^{+}Ly6C^{hi}CD115^{+} cells and CD11b^{+}Gr1^{+}Ly6G^{hi}CD115^{-} cells in BMs, Blood and Spleen. Dot-plots present the representative data from flow cytometry analysis is shown in left and data shown in right. Representative of three independent experiments with three mice per group. (**C**) Tumor-infiltrating CD11b^{+}Gr1^{+} neutrophils were isolated and stimulated with LPS in vitro as indicated to detect the NET. The area of NET is quantified. Representative of three independent experiments with three mice per group. $^{**}P$ <0.05 and $^{***}P$ <0.001, compared with the indicated groups.

Supplementary Fig.6

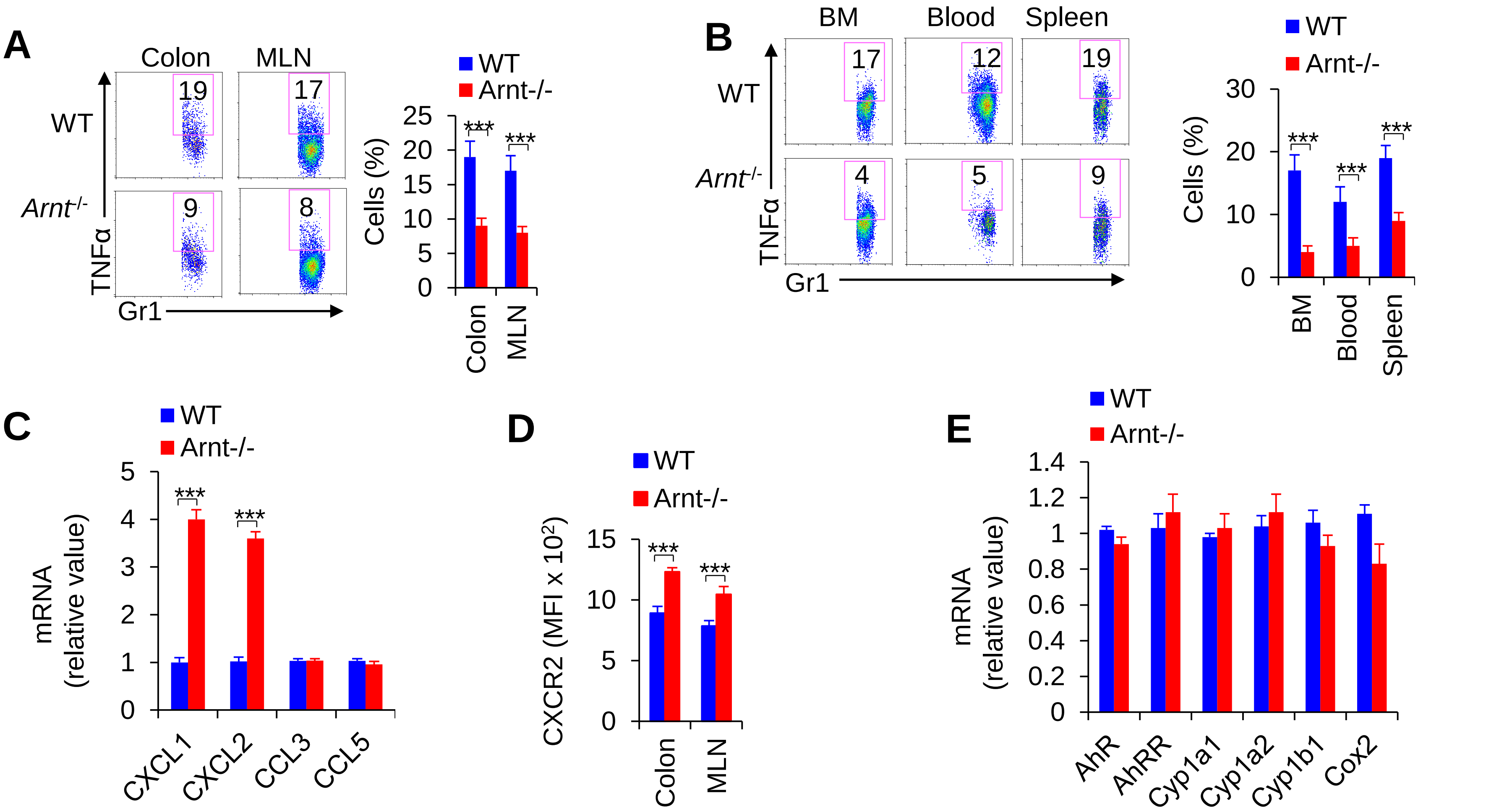

Fig. S6. *Arnt*$^{-/-}$ tumor microenvironment enhances CD11b^{+}Gr1^{+} neutrophil recruitment and functions in promoting the colorectal cancer growth.

Colorectal cancer induction for 80 days after the injection of azoxymethane (AOM) and dextran sulfate sodium salt (DSS) drinking water. (**A-B**) Intracellular staining of TNFα in CD11b^{+}Gr1^{+} neutrophils from colon and MLN (**A**) and BMs, blood and spleen (**B**). Dot-plots present the representative data from flow cytometry analysis is shown in left and data summary shown in right. (**C**) Indicated chemokine mRNA expression of tumor-infiltrating CD11b^{+}Gr1^{+} neutrophils from WT and *Arnt*$^{-/-}$ mice. (**D**) MFI of CXCR2 in CD11b^{+}Gr1^{+} neutrophils from colon and MLN of WT and *Arnt*$^{-/-}$ mice. (**E**) Indicated gene mRNA expression of tumor-infiltrating CD11b^{+}Gr1^{+} neutrophils from WT and *Arnt*$^{-/-}$ mice. Data are representative of three independent experiments with at least three mice per group. ****P* <0.001, compared with the indicated groups.

Supplementary Fig.7

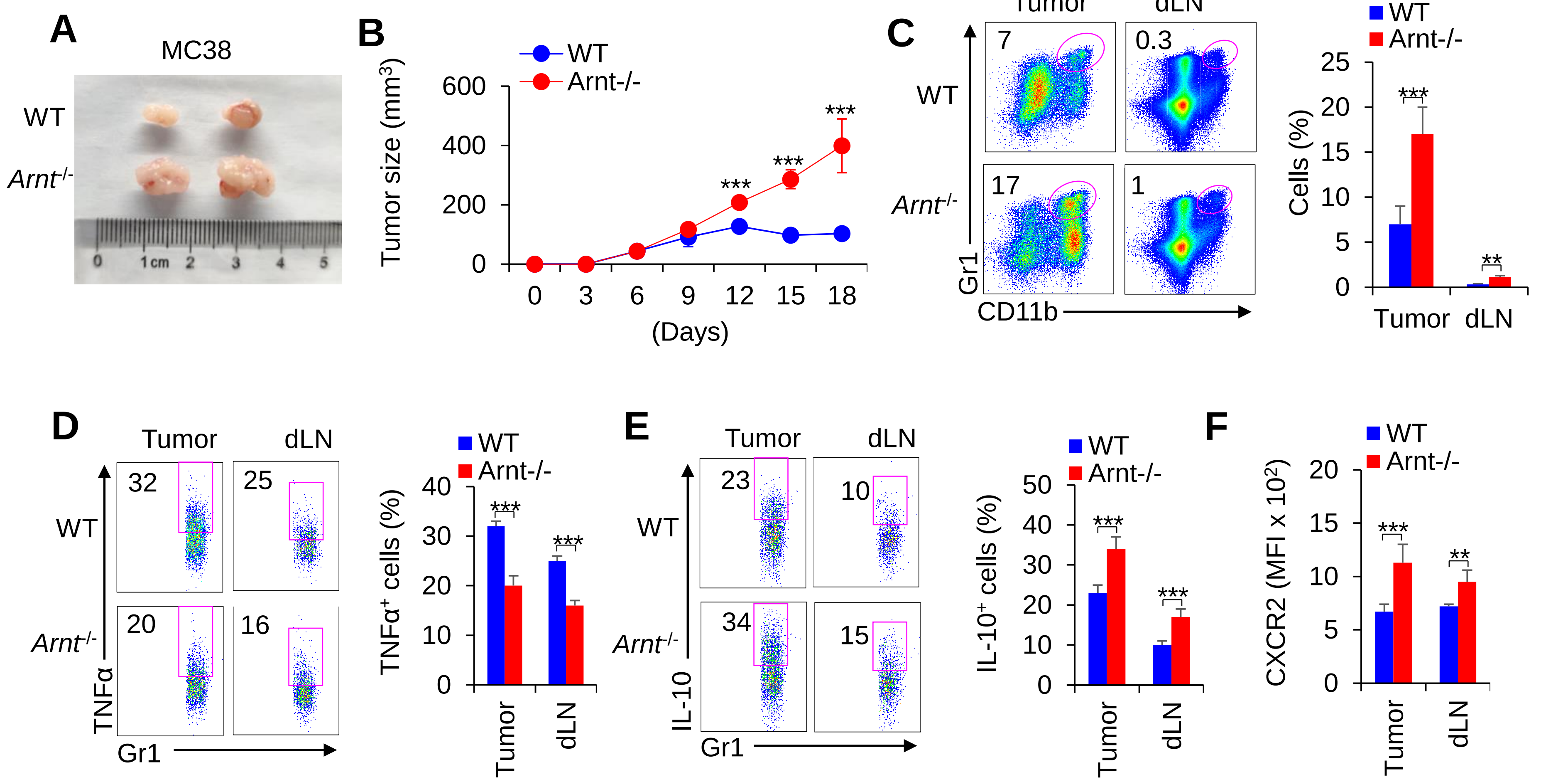

Fig. S7. *Arnt*$^{-/-}$ tumor microenvironment enhances CD11b^{+}Gr1^{+} neutrophil recruitment and functions in promoting the colon cancer growth.

(**A-B**) MC38 tumor cells were implanted subcutaneously in WT and *Arnt*$^{-/-}$ mice (n=10) and tumor growth size was measured every 3 days for 18 days. The representative tumor photo (A) is shown and tumor growth curve (B) are summarized. (**C**) Percent of CD11b^{+}Gr1^{+} neutrophils in tumor and dLN of WT and *Arnt*$^{-/-}$ tumor-bearing mice at day 18. (**D-E**) Intracellular staining of TNFα (D) and IL-10 (E) expression in CD11b^{+}Gr1^{+} cells of tumor and dLN from WT and *Arnt*$^{-/-}$ mice at day 18. Dot-plots present the representative data from flow cytometry analysis is shown on the left, and the data are summarized on the right. (**F**) MFI of CXCR2 in CD11b^{+}Gr1^{+} neutrophils of tumor and dLN from WT and *Arnt*$^{-/-}$ mice by flow cytometry. Representative of three independent experiments with three to four mice per group. **P <0.01 and ***P <0.001, compared with the indicated groups.

Supplementary Fig.8

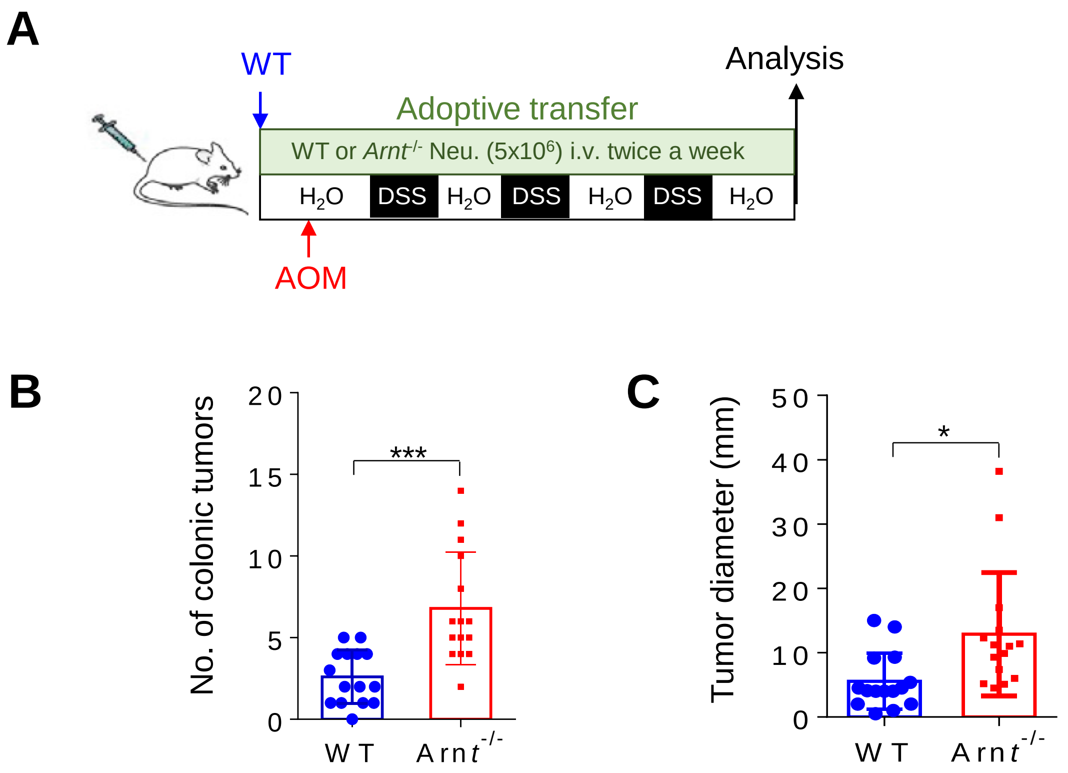

Fig. S8. Tumor microenvironment enhances *Arnt*$^{-/-}$ CD11b^{+}Gr1^{+} neutrophil recruitment and functions in promoting the colorectal cancer growth.

5 × 10^{6} BM neutrophils (Neu.) were isolated from WT or *Arnt*$^{-/-}$ mice every 7 days and transferred to WT recipient mice twice by i.v. injection. The recipient WT mice were injected with azomethane (AOM) and dextran sulfate sodium salt (DSS) drinking water to induce colorectal cancer. (**A**) Carton of experimental program. (**B**) Summary of No. of colonic tumor. (**C**) Summary of tumor diameter. Graph summarizes the data from three independent experiments with fifteen mice per group. *, *P* <0.05 and ****P* <0.001, compared with the indicated groups.

Supplementary Fig.9

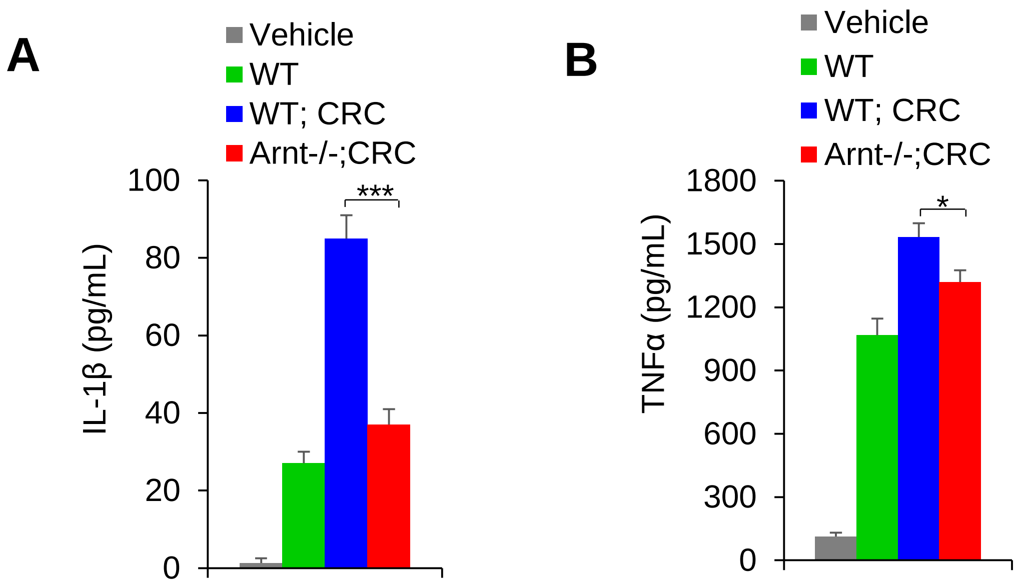

Fig. S9 Analysis fecal metabolites in WT and *Arnt*$^{-/-}$ colorectal cancer mice.
Fecal supernatant was used to stimulate RAW264.7 cell for 3 hrs, and cell culture supernatant were collected and the IL-1β (**A**) and TNFα (**B**) levels were detected in culture supernatant by ELISA. Data are representative of three independent experiments with at least three mice per group. *, *P* <0.05 and ****P* <0.001, compared with the indicated groups.

Supplementary Fig.10

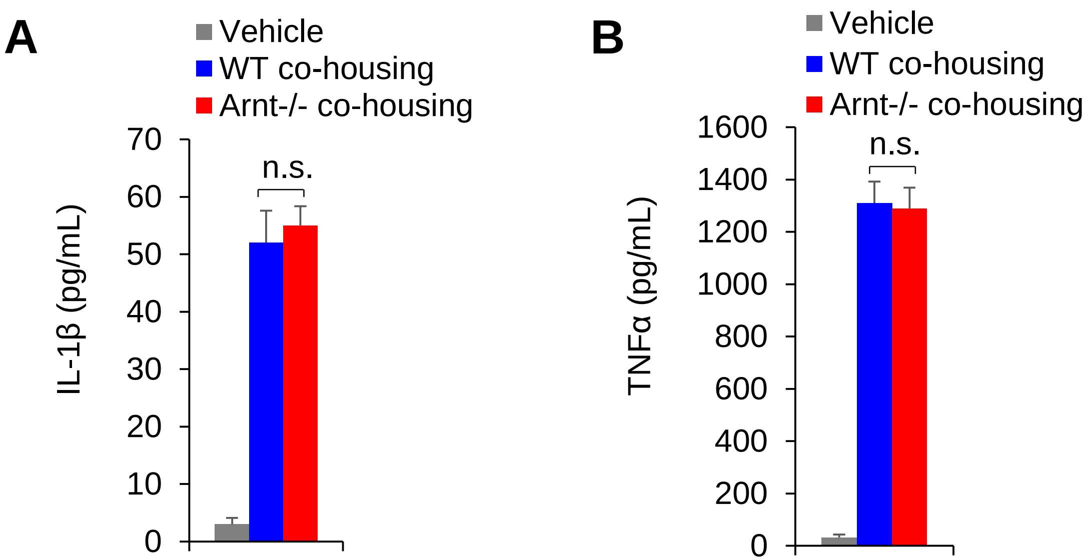

Fig. S10 Analysis fecal metabolites in co-housed WT and *Arnt*$^{-/-}$ mice.
Fecal supernatant was used to stimulate RAW264.7 cell for 3 hrs, and the culture supernatants were collected and the IL-1β (**A**) and TNFα (**B**) levels were detected in culture supernatant by ELISA. Representative of three independent experiments with three mice per group. n.s., not significant.

Supplementary Fig.11

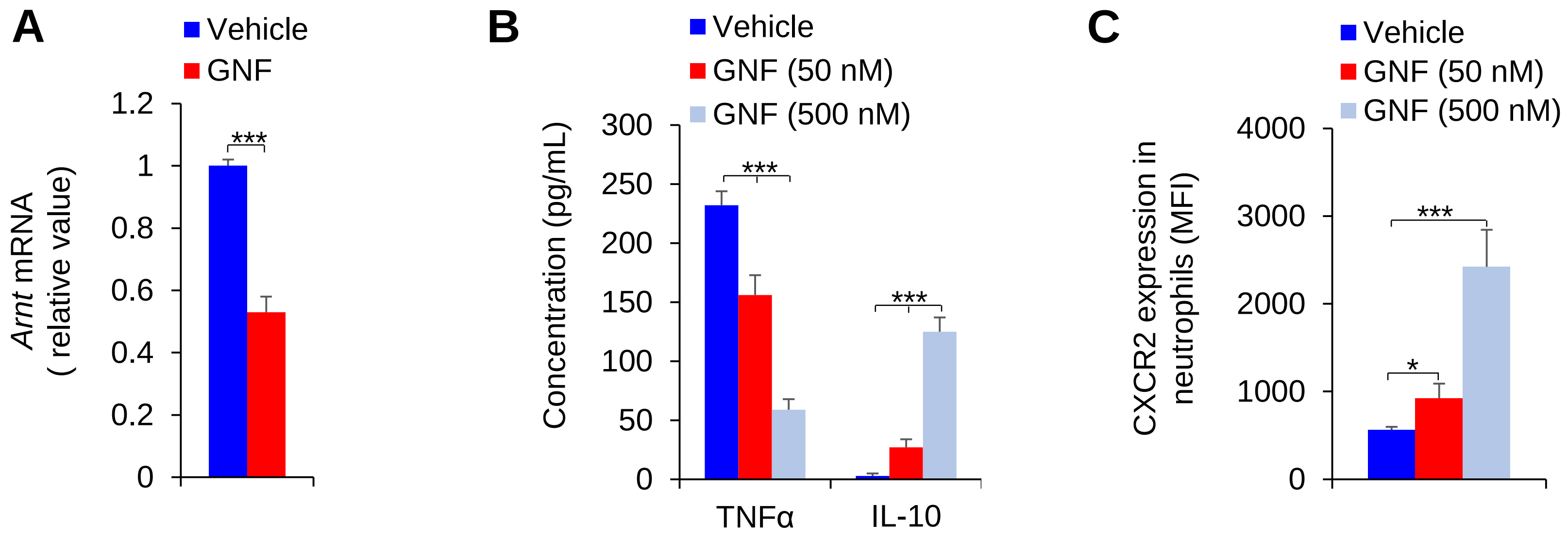

Fig. S11 ARNT regulates the functions of neutrophils in human.

Human neutrophils treated by GNF351 in vitro for 12 hrs. (**A**) mRNA expression of ARNT in neutrophils treated by GNF351 (500 nM). (**B**) Level of TNFα and IL-10 concentration in culture supernatant by ELISA. (**C**) Expression of CXCR2 in neutrophils by flow cytometry. Data are representative of three independent experiments (n=3-4). *, *P*<0.05 and ****P*<0.001, compared with the indicated groups.

Supplementary Fig.12

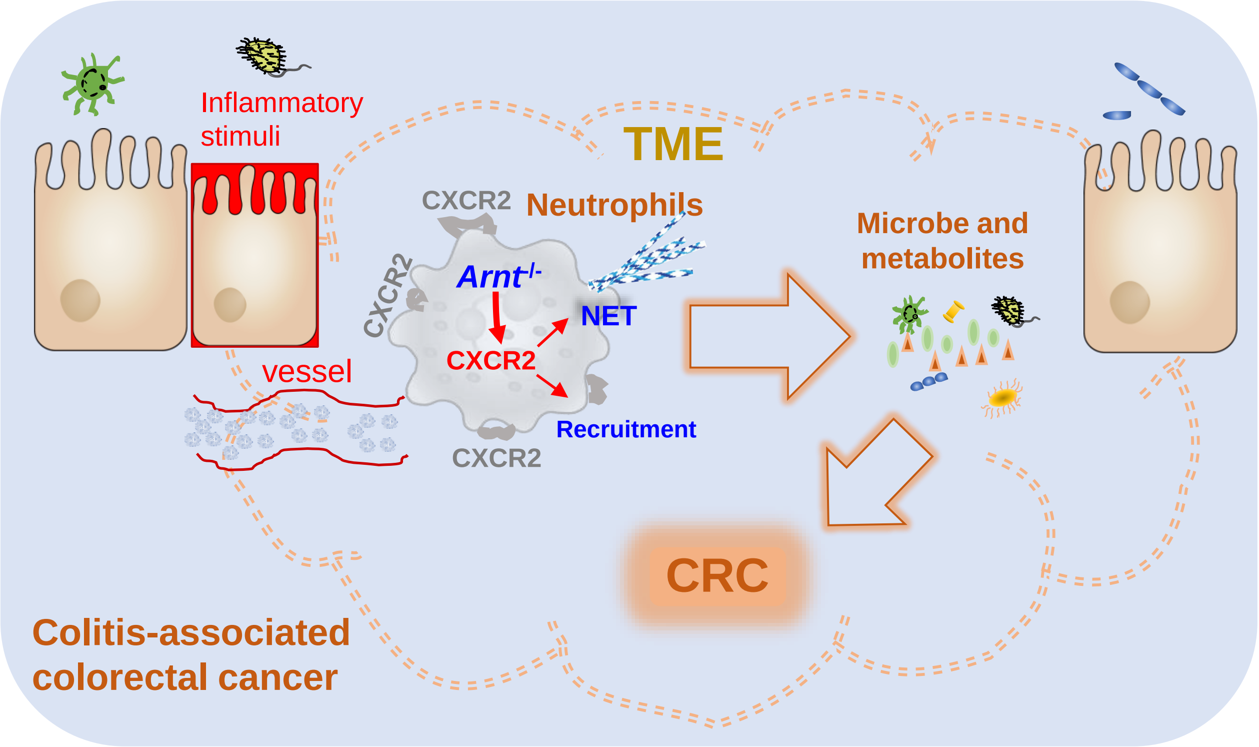

Fig. S12. ARNT limits the recruitment and function of neutrophils in colorectal cancer by regulating gut microbiota. Under inflammatory stimulus, ARNT deletion of $CD11b^+Gr1^+$ neutrophils can significantly up-regulate CXCR2 expression, promote $CD11b^+Gr1^+$ neutrophil recruitment and NET formation, further trigger changes in gut microbiota and metabolites, and play an important role in the anti-colitis-associated colorectal cancer in tumor microenvironment (TME).
